# Betanodavirus and VER Disease: A 30-year Research Review

**DOI:** 10.3390/pathogens9020106

**Published:** 2020-02-09

**Authors:** Isabel Bandín, Sandra Souto

**Affiliations:** Departamento de Microbioloxía e Parasitoloxía-Instituto de Acuicultura, Universidade de Santiago de Compostela, 15782 Santiago de Compostela, Spain; sandra.souto@usc.es

**Keywords:** nervous necrosis virus (NNV), viral encephalopathy and retinopathy (VER), virus–host interaction, epizootiology, diagnostics, control

## Abstract

The outbreaks of viral encephalopathy and retinopathy (VER), caused by nervous necrosis virus (NNV), represent one of the main infectious threats for marine aquaculture worldwide. Since the first description of the disease at the end of the 1980s, a considerable amount of research has gone into understanding the mechanisms involved in fish infection, developing reliable diagnostic methods, and control measures, and several comprehensive reviews have been published to date. This review focuses on host–virus interaction and epidemiological aspects, comprising viral distribution and transmission as well as the continuously increasing host range (177 susceptible marine species and epizootic outbreaks reported in 62 of them), with special emphasis on genotypes and the effect of global warming on NNV infection, but also including the latest findings in the NNV life cycle and virulence as well as diagnostic methods and VER disease control.

## 1. Introduction

Nervous necrosis virus (NNV) is the causative agent of viral encephalopathy and retinopathy (VER), otherwise known as viral nervous necrosis (VNN). The disease was first described at the end of the 1980s in Australia and in the Caribbean [1,2,3], and has since caused a great deal of mortalities and serious economic losses in a variety of reared marine fish species, but also in freshwater species worldwide. 

The causative agent of VER was first described as a “picorna-like virus” [4,5], but after the characterization of a virus purified from diseased larval striped jack (*Pseudocaranx dentex)*, it was considered a new member of the family *Nodaviridae* [6]. This first piscine nodavirus was designated as striped jack nervous necrosis virus (SJNNV). Afterwards, piscine nodaviruses were also identified as the causative agents of VER outbreaks in other fish species, such as European and Asian seabass (*Dicenthrarchus labrax* and *Lates calcarifer*, respectively) [7]. The International Committee on taxonomy of viruses (ICTV) included the piscine nodaviruses within the genus *Betanodavirus* of the family *Nodaviridae* in their seventh report [8], grouping seven species, that were later reduced to the four recognized at present: red-spotted grouper nervous necrosis virus (RGNNV), barfin flounder nervous necrosis virus (BFNNV), tiger puffer nervous necrosis virus (TPNNV) and SJNNV in the eighth report [9]. The first isolation of a betanodavirus was obtained from diseased sea bass using the SSN-1 cell line from whole fry tissue of striped snakehead *Ophicephalus striatus* [10]. 

Over the last 30 years, numerous research articles on betanodaviruses and VER have been published and a considerable amount of knowledge on the disease and causative viruses is available at present. However, further research is needed in terms of virus–host interaction, viral transmission (infection routes, differences in host range among genotypes, viral stability in different environmental conditions…), disease epidemiology (i.e., reservoirs, impact of global warming on the development and spread of the disease…) and infection control in fish farms. In this review, we present the latest findings related to the betanodavirus host range and distribution, with special emphasis on genotypes, host–virus interaction, and VER epidemiology, as well as diagnostics and potential control measures for the disease.

## 2. The Virus

### 2.1. Viral Structure

**Figure 1:**
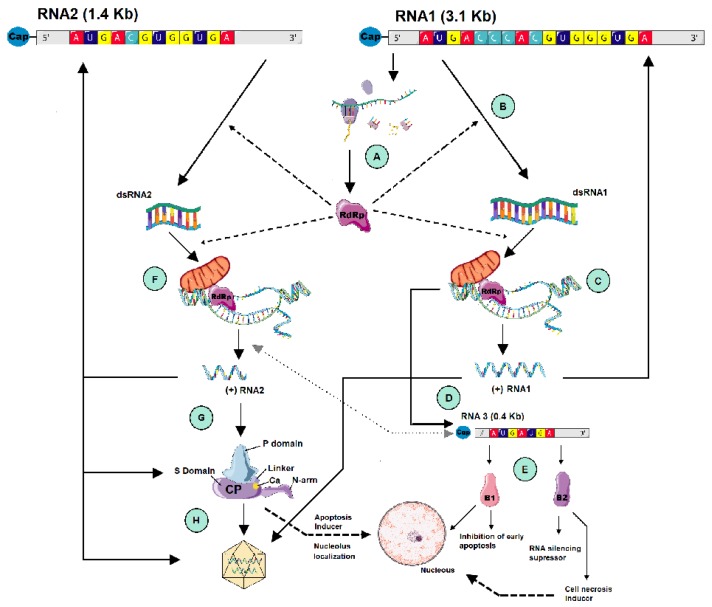
Schematic overview of the betanodavirus replication cycle: After entry, the viral bisegmented single stranded (+) RNA genome is released into the cytoplasm. Subsequently, host ribosomes translate the viral RNA1 into the viral RNA-dependent RNA polymerase (RdRp) (A). The RdRp is then used to copy the genomic (+) RNA1, synthetizing a (−) RNA strand and generating a dsRNA (B). The dsRNA is now used for replication/transcription into new RNA1 molecules (C), all this process takes place in association with outer mitochondrial membranes. Afterwards, a sub-genomic RNA, namely RNA3, is synthesized from the 3’ terminus of RNA1(D). RNA3 encodes -and is translated into- the two small proteins B1 and B2 (E) which show nuclear localization. In addition, RNA3, presumably like in alfanodavirus, also regulates RNA2 synthesis (F) and it is downregulated at the onset of RNA2 replication/transcription (dotted line). RNA2 translation yields the capsid protein (G) and, finally, nascent (+) RNA1 and (+) RNA2 molecules are packaged into progeny virions (H). Adapted from SMART (Servier Medical Art), licensed under a Creative Common Attribution 3.0 Generic License. http://smart.servier.com/.

NNV is a small non-enveloped virus with a diameter of around 25–30 nm and a T = 3 icosahedral symmetry (180 copies of a single protein) [6]. The viral genome is composed of two single-stranded positive-sense RNA molecules known as RNA1 (1.01 × 10^6^ Da) and RNA2 (0.49 × 10^6^ Da), both co-packaged into a single virion (Figure 1). The 5’-ends of these RNAs are capped but their 3’-ends are not polyadenylated. The biggest segment, RNA 1, is composed of around 3100 nucleotides (nt) and contains an open reading frame (ORF) for the RNA-dependent RNA-polymerase (RdRp), also known as protein A. RNA2, the smallest segment (1410–1433 nt), codes for the capsid protein (CP) [6,11]. In addition, a subgenomic RNA, called RNA3 (371–378 nt), which is not packaged into virions, is synthetized from the 3’-end of RNA1 [12,13,14] and codes for two non-structural viral proteins: B1 and B2.

Protein A, one of the three non-structural proteins of the virus, has a molecular weight of 110 kDa and a variable size depending on the viral genotype: 983 amino acids (aa) in SJNNV, 982 aa in RGNNV and 981 aa in BFNNV [12,14,15].

The capsid protein (338 aa, except the CP of SJNNV, which is 2 aa longer), has a molecular weight of 37 kDa [11,15,16,17]. In Alphanodavirus, upon genome encapsidation, the precursor of the capsid protein, protein α, is auto-catalytically cleaved into proteins β and γ [18], generating the mature capsid. This mechanism was not observed in betanodavirus [11]. Instead, the capsid protein undergoes conformational changes which are important for its structure and functions. Intramolecular disulfide bondings between cysteines 187 and 201 [19] or cysteines 115 and 201 [20] have been shown to play a role in the assembly and thermal stability of the viral particles. The structure of the Grouper nervous necrosis virus (GNNV) CP has been disclosed [21] and consists of three different domains and a flexible linker region. The N-terminal arm (N-arm) is responsible for recruiting the RNA during encapsidation; the shell domain (S-domain), a conserved region that forms the cage for the encapsidated RNA and contains calcium-binding structures which seem to be essential for virus assembly [22], and the protrusion domain (P-domain) that includes the hypervariable region of the protein, is involved in the interaction with the host cell surface, and is also responsible for the trimerization of the protein. Besides these structural functions of the CP, it has also been reported to contribute to the modulation of the host cell life cycle during viral infection. A nucleolus localization signal has been identified in the N-terminal region (aa 23 to 31) of the protein, which is associated with cell cycle arrest [23]; at a later stage of infection, the accumulation of the CP triggers apoptosis by inducing a caspase-dependent cascade [24,25].

Although there has been some controversy about the existence of B1 [15,26], this protein, encoded by an ORF that matches the C-terminus of the protein A reading frame, has been demonstrated to play a role as an antinecrotic death factor, which reduces mitochondrial membrane potential (MMP) loss in grouper fin (GF-1) cells [27]. In addition, B1 has been localized in the cytoplasm of E-11 cells infected with an RGNNV strain at 24 hours post infection (hpi) and targeting the nucleus at 48 hpi in up to 95% of cells [28]. The nuclear localization of B1 was mediated by two arginine-rich nuclear targeting domains. This B1 nuclear localization causes cell cycle arrest, confirming its implication in the regulation of host cell survival at the early stages of viral infection in GF-1 cells.

The other ORF is in a +1 reading frame relative to protein A and encodes B2, which is required for the suppression of cellular RNA interference (RNAi) in infected cells [13,29]. B2 has also been identified as a necrotic death factor, which upregulates the expression of the proapoptotic gene Bax and induces MMP loss but not mitochondrial cytochrome c release [30,31]. As with B1, B2 is also localized in the cytoplasm at 24 hpi and in the nucleus at later stages of infection [29,32].

### 2.2. Viral Replication

The genome of positive-strand RNA viruses (+RNA viruses), like NNV, behaves like an mRNA within host cells to allow the expression of viral proteins which are first translated and then amplified by virus-encoded RdRps through negative-strand RNA intermediates. Progeny RNA serves as a template for additional rounds of replication and synthesis of viral proteins [18]. In the case of betanodaviruses, RNA replication is accompanied by the addition of cap structures to the 5’-ends of progeny RNA and the synthesis of a capped subgenomic RNA (sgRNA) derived from RNA1 [13].

RNA is replicated by protein A, which shows a domain spanning aa 582 to 808 and 585 to 819, in RGNNV and SJNNV respectively, containing six of the eight conserved motifs previously identified for RdRps of +RNA viruses [12,15]. Protein A catalyzes RNA synthesis in concert with mitochondrial membranes, mediating the formation of replication complexes with the mitochondria outer membrane (Figure 1). In the related genus *Alphanodavirus*, the N terminus of protein A functions both as a mitochondrial targeting signal and as a transmembrane domain for the tight association of the protein with cellular membranes [18]. Four transmembrane domains (TMDs) with a moderate level of hydrophobicity have been identified for two betanodaviruses, greasy grouper necrosis virus (GGNNV) [33] and Atlantic Halibut Nodavirus (AHNV) [34]. These TMDs are located at positions 1–40, 225–246 for the AHNV, and at positions 153–173, 229–249 for the GGNNV and all but the TMD 153–173 were confirmed to contain mitochondrial targeting signals. In addition, nine amino acid signatures were identified in the sequences of these TMDs at positions 7, 19, 155, 223, 232, 235, 241, 251 and 254 after comparing different genotypes of betanodavirus [35], which could be related to the differences in the growth kinetics of the different genotypes [35]. Recently, one of these positions, amino acid 223, has been reported to probably be involved in NNV thermotolerance [36].

Regulation of the RNA replication depends on cis-acting elements at the 3’and 5’ termini. However, these regulatory elements have not been demonstrated in betanodaviruses yet. In alphanodaviruses, the 3’terminal 50 nt of RNA2 contains a stem-loop structure (3´SL), which acts as a cis-acting replication signal capable of directing the replication of this segment [37]. A putative 3’SL structure has also been predicted in two betanodavirus strains, SJNNV and a reassortant RGNNV/SJNNV [38,39]. However, the role of the 3’SL in the regulation of RNA2 replication in betanodaviruses is not clear [39]. 

RNA replication in alphanodaviruses is also governed by internally located cis acting elements in both RNAs, and RNA 3 is involved in RNA 2 replication, acting as a trans-activactor of RNA2 and suffering a down-regulation at the onset of RNA2 synthesis [18]. Betanodavirus replication studies have suggested that RNA1 is expressed during the early stages of replication, with RNA2 expressed later [40], which would indicate that the betanodavirus cycle is organized into two phases as reported for the related genus alphanodavirus [18]: an early phase, where protein A molecules are synthetized up to a level that ensures the establishment of the replication complexes and a later phase in which capsid protein translation from RNA2 is up-regulated to enable virion packaging. In addition, the RNA1 copy number was significantly higher than that of RNA2 or of NNV infective particles [40]. The role of protein A, codified by RNA1, in the amplification of both genomic strands, would agree with these findings. RNA2 was also detected to a higher extent than the production of infective particles in the first 24 hpi. However, after that, RNA2 and viral infectivity did not differ significantly. These higher RNA2 levels could be due to the different roles of its encoded CP in cell division and apoptosis, as mentioned above.

The NNV replication cycle, as previously mentioned, involves the formation of a dsRNA replication intermediate that would immediately invoke the cellular RNAi response and the destruction of the viral RNA. However, as in its alphanodvirus counterparts, the B2 protein binds to and sequesters dsRNA to protect viral replication intermediates from the cellular RNAi antiviral machinery and allows RNA1 accumulation in the early phase of viral replication [29,41].

### 2.3. Taxonomic Classification 

Based on a small variable sequence of RNA2, namely the T4 region, betanodaviruses have been traditionally classified into four genotypes, which correspond to the species recognised by the ICTV: RGNNV, BFNNV, TPNNV and SJNNV [42]. Three additional genotypes have been proposed, turbot nodavirus (TNV) [43], Atlantic cod nervous necrosis virus (ACNNV) [44] and Korean shellfish nervous necrosis virus (KSNNV) [45]. TNV has been widely accepted as the fifth genotype, although no isolates have been obtained yet; ACNNV, which clusters isolates from Atlantic cod (*Gadus morrhua*), haddock (*Melanogrammus aeglefinus*) and winter flounder (*Pseudopleuronectes americanus)*, has been included as a clade within the BFNNV genotype [46,47,48,49,50], and KSNNV is too recent to have been considered in the literature.

The isolation of nodavirus from other invertebrate hosts such as crustaceans, however, has lead to a reconsideration of the taxonomy of the *Nodaviridae* family and the proposal of a new genus—*Gammanodavirus* [51,52].

An alternative classification scheme of betanodaviruses was proposed by Thiéry et al. [53] which refers to betanodavirus genotypes as numbers (I, II, III and IV, corresponding to RGNNV, BFNNV, TPNNV and SJNNV, respectively) and establishes subgroups within the genotypes. This is the case of the three subgroups (a, b and c) recognized within genotype II (BFNNV), which reflect the genomic differences between the Canadian Atlantic cod (IIa), the Barfin flounder (*Verasper moseri*) (IIb) and the Atlantic halibut (*Hippoglossus hippoglossus*)/French European sea bass (*Dicentrarchus labrax*) isolates (IIc) and the two subgroups within genotype IV (SJNNV), showing the differences between the strains isolated from Senegalese sole (*Solea senegalensis*) and gilthead sea bream (*Sparus aurata*) in the Iberian Peninsula (IVa) and those isolated in Japan (IVb) [53,54].

In recent years, the sequencing of both genomic segments has demonstrated the existence of natural reassortants between the RGNNV and SJNNV genotypes in Southern Europe. Although both combinations of genomic segments, SJNNV/RGNNV and RGNNV/SJNNV (RNA1/RNA2), have been observed in viral isolates obtained from fish, the second type has been detected more often [55,56,57,58].

Serological studies have demonstrated that the four genotypes can be grouped into three distinct serotypes [59,60]; however, a different correlation between serogroups and genotypes has been proposed. Thus, whereas according to Mori et al. [59] serogroup A would correspond to genotype SJNNV, group B to TPNNV and group C to RGNNV and BFNNV, Panzarin et al. [60] establish that serogroup B would include strains from genotypes BFNNV and TPNNV and that only RGNNV strains would be clustered in serogroup C. 

### 2.4. Geographical Distribution and Host Range 

**Figure 2:**
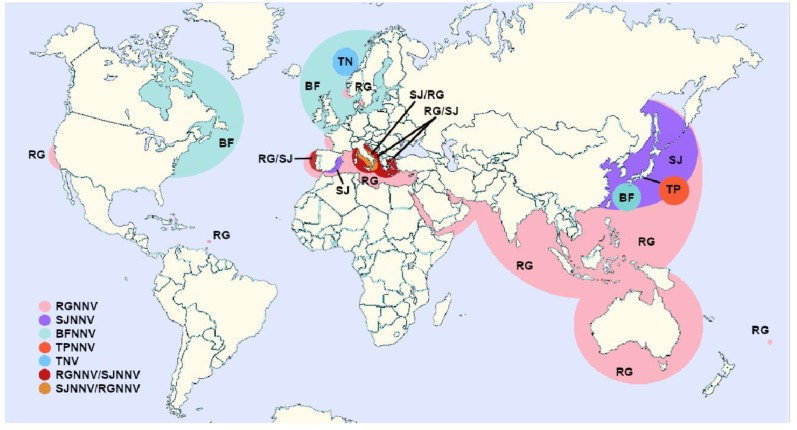
Distribution of Nervous necrosis virus (NNV) genotypes. Adapted from SMART (Servier Medical Art), licensed under a Creative Common Attribution 3.0 Generic License. http://smart.servier.com/.

Betanodaviruses are widely distributed (Figure 2), but the geographical distribution of the different genotypes seems to be related to their thermotolerance. As a result, the BFNNV genotype seems limited to cold-water fish in Japan and Northern areas of Europe and America (Figure 2, Table 1, Table 2, Table 3 and Table 4). It has been mainly isolated from farmed fish, including Atlantic and Pacific cod (*G. macrocephalus*), haddock (*Melanogrammus aeglefinus*), Atlantic halibut and barfin flounder; but it has also been detected in some wild species, such as Atlantic cod and different species of wrasse (ballan, corking and goldsinny wrasse, *Labrus bergylta, Symphodus melops* and *Ctenolabrus rupestri*) in Scandinavian coastal waters [47,50] and winter flounder (*Pseudopleuronectes americanus*) in Canada [61].

The RGNNV genotype, which affects tropical and temperate fish species, is the most widely distributed (Figure 2) and has the highest number of susceptible species (Table 1, Table 2 and Table 4). In recent years, surveys conducted in different geographical areas have shown that RGNNV is widely distributed not only in farmed but also among wild fish in the Mediterranean basin and along the coasts of Asia and Australia [46,62,63,64,65,66,67,68,69,70,71,72]. It has also been isolated from farmed white seabass (*Atractoscion nobilis*) in California [73]. In addition, it has recently been reported in wild ballan and corkwing wrasse inhabiting Scandinavian waters [50], which are usually associated to the BFNNV genotype. Finally, it is the only genotype associated to outbreaks in freshwater species in Europe, Asia and Australia (Table 3).

In contrast, the TPNNV genotype seems to be a minor variant because it has only been described in one species in Japan [42].

The SJNNV type, although for a long time considered limited to fish reared in Japanese waters, has also been detected in Senegalese sole and gilthead sea bream farmed on the Iberian Peninsula [54]. 

Finally, natural SJNNV/RGNNV reassortants have only been isolated from sea bass on the Italian coast [95], whereas the opposite form, RGNNV/SJNNV, is widespread in Southern Europe (Figure 2) and has been isolated from farmed European sea bass, sole (both common sole, *S. solea*, and Senegalese sole) and gilthead sea bream [55,56,57] and recently from wild Mediterranean horse mackerel (*Trachurus mediterraneus*) [58].

### 2.5. Viral Thermotolerance

The risk of infectious disease outbreaks in the aquatic environment depends on the interactions between host and pathogen, as well as environmental factors. Among these, temperature has a crucial effect on viruses hosted by fish, because these animals have virtually no capacity to maintain a difference between their body temperature and that of the environment. Temperature can modulate the ability of the fish to defend itself against infection but also the ability of the infectious agent to colonize the host fish. This effect is especially evident in betanodaviruses because betanodavirus genotypes show different optimal growth temperatures (15–20 °C for BFNNV, 20 °C for TPNNV, 20–25 °C for SJNNV and 25–30 °C for RGNNV) and therefore, natural infections can occur at different water temperatures depending on the viral type. Thus, whereas BFNNV has been reported to cause disease at temperatures as low as 4 to 15 °C [47,144], NNV outbreaks associated to RGNNV are linked to high water temperatures: from 23 to 30 °C (in sea bass) to 28 to 30 °C (in different grouper species) [144,145]. Experimental trials have also demonstrated the effect of temperature on NNV pathogenicity [145,146,147,148,149,150].

The temperature sensitivity of betanodaviruses seems to be regulated by RNA1 [35,151] and more specifically by the region encoding the amino acid residues 1–445 [151]. Experiments performed with a recombinant strain harboring six point mutations in this region confirmed its role in viral thermotolerance, and pointed to position 223 as a putative responsible for temperature regulation [36]. This study, however, showed that genomic regions other than 1–445 may be involved in NNV thermotolerance.

Global warming is causing serious modifications in aquatic environment parameters, including changes in dissolved oxygen and ocean carbon dioxide levels, salinity and temperature. It is well documented that changes experienced by ecosystems will affect the epidemiology of infectious diseases in animals, in the wild and under intensive farming conditions, including aquaculture [152,153,154,155,156]. The high number of wild asymptomatic species infected with NNV, mainly in Asian and Mediterranean countries [62,64,66,67,68,71,80,82] and the quick rise in the viral load and subsequent outbreak of mortalities observed in experimentally infected sole when water temperature increased from 16 to 22 °C [148], suggest that VER outbreaks could have dramatic effects on natural populations, as has already been reported for some endangered fish species in the Mediterranean [72,96,109]. On the other hand, it could be argued that ocean warming could decrease the pathologies associated with the BFNNV genotype. However, as indicated above, this genotype can cause disease in a range of temperatures from 4 to 15°C. Furthermore, the number of susceptible species to this genotype could also increase by the movement of some fish species to higher latitudes because of the warming of their natural habitats.

## 3. The Disease: Viral Encephalopathy and Retinopathy (VER) or Viral Nervous Necrosis (VNN)

Disease outbreaks have been reported mainly in early developmental stages (larval and juveniles), but significant mortalities have also been described in older fish [74]. Although clinical signs depend on the fish species, biological stage, phase of the disease and temperature, abnormal swimming behavior (spiral swimming, whirling, horizontal looping or darting) and loss of appetite are commonly observed among affected fish. Other signs include swim bladder hyperinflation and coloration abnormalities (pale or dark). 

Histopathological analysis reveals extensive necrosis of the central nervous system (CNS), with extensive vacuolation and neural degeneration of the brain as well as vacuolation of the retina [5,94,157]. In addition to histological lesions in nervous tissues, hyperplasia with vacuolar degeneration of epithelial cells has been reported in the epithelial layer of the skin, gill operculum, and oral cavity in 1- and 2-day-old striped jack larvae [157]. There also seem to be histological differences according to fish size, because affected larval neural tissue showed a greater extent of vacuolation and in different brain areas (medulla oblongata and spinal cord) than adult fish, which showed lesions in peripheral layers of the molecular layer [158].

### 3.1. Routes of Infection and Spread through the Fish Body 

Several portals of viral entry have been suggested, including epithelial cells covering the fish body and/or the fin [159], gills [159,160,161] and nasal and oral cavity [158,159,160,162].

The neurotropism of betanodaviruses has been repeatedly demonstrated (see reviews [74,144,163]) and viral replication seems to be almost entirely restricted to nerve tissue, preferentially brain and retina [157,162,164]. Histopathological studies have demonstrated the vacuolation of nerve cells in the olfactory lobe and cerebellum (Purkinje cell layer and the underlying granule cell layer) [10,82,119,120,125,135,161,165,166]. Megalocells and small nerve cell nuclei were also infected in the preoptic area, thalamus, medulla oblongata and spinal cord, whereas only a few small nerve cells were infected in the olfactory bulb and optic tectum [57,93,109,119,158,166,167,168].

To date, it has not been possible to identify the neuronal receptors involved in NNV entry and the available data on cell receptors comes from studies using cell lines. Viral entry is believed to occur through clathrin-mediated endocytosis [169] and interaction with different cell receptors has been proposed. Consequently, sialic acid seems to be involved in NNV binding to SNN-1 cells [170] and therefore also to E-11, which are a clone of SSN-1 [171]. In grouper fin cells (GF-1), the grouper heat shock cognate protein 70 (GHSC70) has been proposed as an NNV receptor or co-receptor protein [172] and in SB cells, receptors have been reported to probably be proteins located at lipid rafts or even specific lipids [169]. Recently, Nectin-4/PVRL4, belonging to the family of immunoglobulin-like cell adhesion molecules, has been identified as a potential cellular receptor for NNV in both seven-band grouper and transfected SSN-1 cells [173,174]. In the study performed with the seven-band grouper nectins, a possible interaction with NNV was predicted based on a docking simulation, and interaction with Nectin-4 was the most highly supported [173]. A subsequent assay performed on SSN-1 cells transfected with Nectin-4 indicated that the overexpression of this protein enhanced viral replication kinetics, whereas silencing reduced virus–cell interaction [174].

The C-terminal region of the NNV capsid protein, located in the P-domain [21], has been reported to be involved in host specificity [175,176] and positions 247 and 270 have been identified as putative receptor binding sites, because their substitution modifies the affinity of the virus for the neural receptors and affects the kinetics of the virus spreading in the brain [177]. 

Viral spreading is thought to be produced through the nervous system across the synaptic connections [146,178]. However, NNV has also been detected in blood samples of Senegalese sole, Atlantic cod and seven-band grouper [179,180,181], suggesting that the virus can also use the hematogenous route to spread throughout the fish body. Viral presence in non-neurological tissues (gills, fins, heart, anterior and posterior intestine, stomach, spleen, liver, kidney and gonads) [158,180,182,183,184,185,186] would support hematogenous spread. However, as all these organs are fully innervated, neural spread cannot be ruled out. Analyses of experimentally infected Atlantic cod suggest an initial viremia followed by neural spread [180]. Although in most cases NNV detection in non-neurologicaltissues has been performed using histological or molecular techniques, the presence of infective particles has been confirmed in gills, skin, fins and intestine of Senegalese sole [159] and testis of European sea bass and gilthead sea bream [187].

The dynamics of NNV infections seem to be fast; the RGNNV genome was detected in the brain of infected pompano at 4 hpi [188] and both genome and infective particles of a reassortant RGNNV/SJNNV strain were detected after 1 day post infection (dpi) in the brain of Senegalese sole [159]. However, differences related to genotypes and fish species can be observed because in Senegalese sole the RGNNV genome was not detected until 3 dpi, and the detection of infective particles took one more day (4 dpi) [159]. This fast replication also implies the fast development of disease signs in infected fish, which were observed as early as 2 dpi in intramuscularly injected juvenile European sea bass and bath infected Asian sea bass larvae [160,189]. In both cases, first mortalities were detected soon after the onset of clinical signs. In other intramuscular experimental infections of European sea bass, first disease signs were recorded after 4–5 dpi [164,185]. Clinical signs were also soon observed in bath infected Senegalese sole (3 dpi) [190] and slightly later in bath infected European sea bass (5 to 10 dpi) [145,147,160]. The onset of mortality was detected very soon in bath-infected striped jack larvae (3–4 dpi) [70] and at 5 dpi in Senegalese sole, reaching 100% mortality at 18 dpi [190].

### 3.2. Viral Transmission

Both horizontal and vertical transmission have been demonstrated in several fish species. Horizontal transmission, fish to fish or through the water body, has been reported in Asian sea bass or barramundi, European sea bass, gilthead sea bream, brown-marbled grouper (*Epinephelus fuscoguttatus*) and Senegalese sole [5,92,148,160,164,191,192,193,194]. Similarly, interspecies horizontal transmission has been observed between European sea bass and gilthead sea bream reared on the same farm [129] and in experimental trials between turbot and Atlantic cod [195] and between Asian seabass and brown-marbled grouper [194]. Viral shedding from gills and skin could be involved in this transmission, as reported in Senegalese sole [159]. The main factors affecting horizontal transmission in farming conditions are stocking density [196], and temperature [148,149]. Genotype also seems to be an important factor, as shown by different challenge experiments, i.e., reassortant RGNNV/SJNNV and SJNNV strains isolated from Senegalese sole caused low mortalities in European sea bass through bath challenges [145,190].

Invertebrates, mainly bivalve mollusks (mussel, clam and oyster) but also gastropods and some crustaceans (crab, shrimp and lobster species) (Table 5) can act as natural reservoirs and possible carriers of NNV and, therefore, play a role in viral transmission through the water column, which can be favored by NNV resistance to environmental conditions and long survival in sea water [10,197]. In addition, two crustacean species, brine shrimp (*Artemia salina*) and rotifer (*Brachionus plicatilis*), used as live food for marine fish larvae, have been demonstrated to be susceptible to NNV infection [198] and in the case of Artemia, capable of transmitting NNV to Senegalese sole larvae causing disease symptoms and high mortality [199].

Vertical transmission has been reported in striped jack [182,203], Japanese flounder (*Paralichthys olivaceus*), barfin flounder [204], Atlantic halibut [146], European and Asian sea bass [183,205]. Gonads have been demonstrated to be involved in viral shedding in striped jack, European sea bass and gilthead sea bream [182,187,203,205]. In striped jack and Senegalese sole, the intestine has also been reported to take part in viral release [159,182] which would result in the contamination of eggs [182].

Both ways of transmission are a serious concern for the fish farming industry. The detection of carriers among breeders, farmed population and fish to be introduced into aquaculture sites is at present one of the major strategies to control disease outbreaks [126,179,206,207,208,209]. 

### 3.3. Host Response

NNV infection in fish provokes a host immune response, which is not completely understood yet, but an excellent review on the subject has already been published [163]. Briefly, although fish resistance to viral infections is mediated by innate and adaptative response, the first one seems to play a relevant role [210]. Innate immunity represents the first antiviral defense and is mediated by interferon (IFN) and interferon-induced genes (ISGs).

To date, three types of IFN have been described in vertebrates (type I, II and III). Types I and II-IFN are present in fish [211,212] and both have been detected in individuals infected with betanodavirus [163]. I-IFN transcription has been reported to be up-regulated in sea bass [213] and grouper [212], II-IFN production increased in experimentally infected turbot [214], whereas both I and II IFN were induced in infected zebra fish [215]. IFN up-regulation has also been described in *in vitro* assays, in barramundi brain (BB), grouper brain (GB) and FHM cells [216,217,218]. The up-regulation of interferon regulatory factors (IRF) and ISGs has also been reported in different species: IRF1 in infected turbot, Mx in gilthead sea bream and European sea bass [213,219,220], ISG-12 in European sea bass [221] and ISG-15 also in European sea bass and Senegalese sole [220,222]. In addition, the induction of other pro-inflammatory cytokines has been analyzed; as a result, the increased expression of tumor necrosis factor α (TNFα) has been reported in gilthead sea bream and sea bass [219,221], Interleukin 1-β (IL-β) is up-regulated in gilthead sea bream [219] and IL-β together with IL-1 and IL-34 is over-expressed in golden pompano (*Trachinotus ovatus*) [223]. 

Regarding the cell immune response to betanodavirus infection, an increase in the expression of T-cell marker genes (TRCb, CD4-2, CD4, CD8a, CB8b, Lck, NCCRP-1 and ZAP-70) has been reported in infected Atlantic halibut, European sea bass and gilthead sea bream [213,214,224,225]. In addition, the proliferation of CD4-1-positive lymphocytes has been assessed by flow cytometry in infected olive flounder (*Paralichthys olivaceus*) [226]. 

In recent years, a great deal of progress has been made on high-throughput tools for sequencing the transcriptome (RNA-Seq), enabling genome-wide transcriptomic analysis and providing valuable information for understanding virus–host interactions [227]. Different transcriptomic analyses have been performed on both NNV-infected cells [228,229,230,231,232,233] and fish [234,235,236,237,238].

The transcriptome analyses of different cell lines susceptible to NNV have provided useful information about the immune response elicited against viral infection. In SSN-1 cells infected with a RGNNV strain, the down-regulation of 1138 genes and the up-regulation of 2073 involved in different pathways related to viral pathogenesis was observed. Subsequent analyses focusing on the apoptosis pathway showed an over-expression of Endonuclease G, which could be responsible for cellular apoptosis [230]. RNA-seq analyses of *D. labrax* brain (DLB-1) cells also infected with an RGNNV strain, showed the up-regulation of a high number of genes related to immunity, heat-shock proteins or apoptosis. Gene ontology enrichment revealed the down-regulation of transcripts related to the cytoskeleton and vesicle biology, suggesting that the failure of vesicle transport upon NNV infection could be a major mechanism behind the pathogenic effects on the fish nervous system [232]. In another study also performed with DLB-1 cells, the transcriptomic profiles obtained from European sea bass head-kidney leucocytes incubated with NNV infected and uninfected cells were very similar, supporting that cell-mediated cytotoxic activity in sea bass is not primed upon NNV infection [231]. Other cells used for transcriptome analysis were Asian seabass (*Lateolabrax japonicus*) epithelial cells (SB) [229], grouper kidney (GK) cells [228] and *Lateolabrax japonicus* brain cells (LJB) [233]. The assembly of the transcriptome of NNV-infected SB cells, as in the previously described cell lines, showed a strong induction of various genes relevant to innate immunity which were identified as receptor-transporting 3 (RTP3), Viperin, IRF3, IFN and two heat shock protein (HSP) family members (Hsp30 and 70) [229]. In the NNV-infected GK cells, 117 genes associated with protein processing in endoplasmic reticulum (ER) were identified. In addition, the tag-based digital gene expression (DGE) system revealed that ER stress response was clearly affected in NNV-infected GK cells. A further analysis revealed an interaction between the NNV capsid protein and the ER chaperone immunoglobulin heavy-chain binding protein (BiP), suggesting that the capsid protein plays a role in the NNV-induced ER stress [228]. Finally, in LJB infected with an RGNNV strain, 1969 up-regulated genes and 9858 down-regulated genes involved in immune response pathways were identified. It was also observed that the p53 signaling pathway was involved in NNV infection and inhibited by RGNNV. The overexpression of *L. japonicus* p53 (Ljp53) significantly inhibited RGNNV replication and up-regulated the expression of apoptosis-related genes, suggesting that Ljp53 might promote cell apoptosis to repress virus replication [233].

Regarding transcriptome analysis in infected fish, several studies have been performed in grouper species. Thus, a significant up-regulation of antiviral proteins and NK-Lysin, a known antibacterial protein, was observed in the brain of NNV infected sevenband grouper (*E. septemfaciatus*). Furthermore, several chemokines, cathepsins and lepsins were also up-regulated [239]. Brain tissue was also analyzed in persistently infected Malabar grouper (*E. malabaricus*), showing that highly immune cell active signaling and surface receptor expression were triggered during persistent infection, as well as the interferon-induced response [236]. Therefore, although immune cell activity was high in brain tissue during persistent infection, this failed to eliminate all the viral particles from the infected host. Further examination of the impaired virus clearance pathway revealed the up-regulation of some genes involved in immune cell suppression, such as PDL1 and LAG3, which are considered critical markers for persistent and chronic infection [240,241]. A different study analyzed the transcriptome of kelp grouper (*E. moara*) immune tissues (liver, spleen and kidney) and although the expression of class I major histocompatibility complex (MHC) was significantly higher in three immune tissues of the diseased grouper, many immune related genes, including humoral immune molecules (such as antibodies), the cellular mediated cytotoxic molecules (such as perforin) and some adhesion related genes were down regulated [238].

In acute nodavirus infections, there must be a balance of induction and inhibition of immune responses [212], and nodaviruses must be able to evade the host’s protective systems so that they can replicate and transmit progeny to other cells. However, it is worth mentioning that the immune response may contribute to disease signs and mortality, as shown in experimental infections in Senegalese sole performed with an RGNNV/SJNNV reassortant strain, highly virulent for this species, (wt), and an attenuated mutant strain showing two amino acid changes in the capsid, which caused a 40% mortality decrease [237]. In this study, a higher number of genes (633) were differentially expressed (DEGs) in animals infected with the highly virulent wt isolate, when compared with animals infected with the mutant strain (393). In addition, in the eye/brain samples the proportion between up-downregulated DEGs was 91% and 9% after infection with the wt isolate, whereas the proportion was completely inverted in fish infected with the mutant strain (11% and 89%). This result was corroborated in an experimental infection performed on sea bass with an RGNNV recombinant strain harboring the same mutations in the capsid protein [221] because low or no inflammatory induction (transcription of *mxA*, *isg 15* and *tnf alpha* genes) was observed in the brain of fish infected with the mutant strain, whereas a strong induction was observed in fish challenged with the wt isolate. Finally, a similar result was observed in primary cultures of grouper brain cells, where NNV infection may activate microglial proliferation and stimulate microglial secretion of interleukin (IL)-1b, which is a critical cytokine responsible for neuronal death [242].

On the other hand, it has been observed that some fish are resistant to infection with a certain genotype, but susceptible to infection with a different viral type, i.e., gilthead sea bream was long considered an asymptomatic carrier of RGNNV strains [129] and it has recently been reported to be highly susceptible to RGNNV/SJNNV reassortants [57]; in a similar way, an RGNNV isolate was also obtained from asymptomatic turbot and did not produce mortalities in experimental challenges [132], whereas this fish species undergoes high mortalities associated with TNV genotype [43]. However, to date, no studies have been performed to identify the factors involved in the susceptibility/resistance of these species to different viral types.

### 3.4. Disease Outbreaks 

It is well known that a disease outbreak is influenced by three different parameters: the environment, the host and the pathogen [243]. Different environmental factors have been postulated as predisposing factors for VER outbreaks, including temperature, stocking density and stress [74]. As mentioned above, host response is being thoroughly studied, although the differential susceptibility to genotypes has not yet been analyzed. On the other hand, only a few reports have been focused on NNV virulence determinants. The fact that most NNV isolates obtained from farmed fish have caused high mortalities [5,72,92,97,102,137], and therefore can all be considered highly virulent strains, and the scarce number of isolates obtained from asymptomatic reared fish, which could be considered as avirulent, probably explains the low number of studies on NNV virulence. However, the increasing number of detections in farmed and wild asymptomatic fish [58,62,63,64,65,66,68,126,130,132,201] should also increase the number of isolates which could be used in the future for virulence studies.

Some recent reports have focused on the C-terminal region of the capsid protein, as it is involved in host cell recognition [175,176]. Studies performed with a reassortant RGNNV/SJNNV strain demonstrated that the substitution of amino acids 247 (Ser→Ala) and 270 (Ser→Asp) in the SJNNV-type capsid brought about a 40% reduction in virulence in sole [244] and reduced viral replication in sole neurons [177]. The modification of these positions in an RGNNV strain also resulted in a sharp decrease in mortality in infected sea bass [221], which confirms that these two positions are involved in NNV virulence, regardless of the viral genotype. Other studies have also indicated that substitutions in the 3`terminal end of RNA2 lead to an attenuation in virulence for Senegalese sole and a delayed replication in brain tissues which could be due to the interaction of RNA2 with host cellular proteins [39].

## 4. Epidemiology

### 4.1. VER Outbreaks and NNV Detections in Farmed Fish

Since the first descriptions in the 1990s, VER episodes have been constantly reported, mainly in marine fish reared in Asian, Australian and European waters [57,74,86,90,91,115,117,120,245]. The most important affected species include grouper, Asian seabass/barramundi, European sea bass, gilthead sea bream, Japanese and barfin flounder, Atlantic and Pacific cod and Atlantic halibut (Table 1). However, routine surveys conducted on farmed fish have revealed the existence of a great number of asymptomatic individuals which could act as carriers. Therefore, NNV asymptomatic carriers have been detected among farmed species that have been reported to undergo disease outbreaks, such as European sea bass, golden pompano, Japanese parrotfish (*Oplegnathus fasciatus*), Japanese flounder (*Paralichthys olivaceus*), different grouper species (*E. lanceolatus*, *E. akaara* and *E. awooara*), red drum (*Sciaenops ocellatus*), dusky sinefoot (*Siganus fuscescens*) and tiger puffer (*Takifugu rubripes*) [56,66,68,131]. In all these cases, except the detection in Japanese parrot fish, which was not typed, viruses causing non-clinical infection belonged to the RGNNV genotype. Furthermore, the RGNNV genotype, as described above, has been isolated from asymptomatic farmed fish which have suffered clinical infection caused by a different genotype, i.e., turbot [132] and gilthead sea bream [63]. The RGNNV genotype has also been detected by PCR in other reared fish species in Asian countries which have not suffered VER outbreaks to date [65,66,67,68,69] (Table 2).

All these reports suggest a high prevalence of the RGNNV genotype in farmed fish in Asian countries, but also in the Mediterranean basin, which can lead to disease outbreaks when temperature and/or fish density increases.

Finally, RGNNV strains have also caused most of the outbreaks in freshwater species. [17,46,49,84,135,137] (Table 3) and have been detected in ornamental fish, both marine and freshwater species (Table 2). It is worth noting the viral presence in two species native to the Amazon river, South American leaf fish (*Monocirrhus polyacanthus*) and red piranha (*Pygocentrus nattereri*) [126,246] because no reports of NNV have been carried out in South America, but the authors conclude that the infection most probably occurred in a Korean aquarium. 

### 4.2. NNV in Wild Fish

In recent years VER outbreaks have also been reported in wild fish in different geographical areas (Table 1). Mortalities associated to NNV have been recorded in wild grouper *(E. costae* and *E. marginatus*) inhabiting European and African Mediterranean waters [72,96,106,109], in European sea bass also in the Mediterranean basin [96], in mullet (*Liza aurata* and *L. saliens*) in the Caspian sea [82] and in milky fish (*Chanos chanos*) in the Indian ocean [80]. In addition, different surveys, mainly in Asian and European waters, have reported the detection of NNV in a wide variety of asymptomatic fish belonging to more than 120 different species, 54 families and 19 orders [50,58,62,63,64,65,66,67,68,69,126,130,139,140] (Table 4). Around 90% of these detections have been genotyped and 86% have been clustered with the RGNNV genotype. SJNNV was also detected in 5.5% of those species, whereas BFNNV was only present in 1.8% of the RGNNV positive species (Table 4). All these data demonstrate the high prevalence of the RGNNV genotype among wild fish and the threat that it represents for these populations, some of them endangered ones [72]. The SJNNV genotype alone was detected in wild European eel in the Albufera lake (Spain) [138] and BFNNV in Atlantic cod and goldsinny wrasse [47,50], both in the Scandinavian peninsula. Similarly, a reassortant strain (RGNNV/SJNNV) has been detected in Mediterranean horse mackerel in Greece [58].

### 4.3. NNV in Invertebrates and other Marine Animals

NNV has been detected so far in 21 species of marine invertebrates belonging to 12 families and nine orders (Table 5). Most of these detections have been performed in bivalve mollusks, which can accumulate different particles, including viruses, from the surrounding water due their filter-feeding activity [200,247]. However, NNV has also been detected in cephalopods such as octopus and squid, [202] crustaceans [126,127,201] and gasteropods [58]. The genotype most frequently detected among invertebrates has been RGNNV, although BFNNV strains have also been reported in single detections or accompanying RGNNV strains in Asian waters. In addition, other mollusk isolates obtained in Asia have been clustered in a new proposed genotype, KSNNV.

Recently, the first betanodavirus isolation from a marine vertebrate other than fish has been reported from a loggerhead turtle (*Caretta caretta*) in Italy [248], widening the range of susceptible hosts for the virus. The animals did not show evidence of disease caused by NNV, which suggests their role as carriers.

## 5. Diagnostics

### 5.1. Cell Cultures 

Isolation in cell culture represents a basic tool for a comprehensive study of any viral agent. Although different fish cell lines, including RTG-2, CHSE-214, BF2, SBL, FHM and EPC, were tested for susceptibility to NNV when the disease viral etiology was confirmed [4,6,11,111,249], the first successful isolation of a betanodavirus was not achieved until the SSN-1 cell line, established from whole fry tissue of striped snakehead *Ophicephalus striatus* [10] was used. Subsequently, the GF-1 cell-line derived from grouper *Epinephelus coioides*, E-11, a clonal line derived from SSN-1 cells, and SB derived from Asian sea bass, were also demonstrated to be useful for the isolation and proliferation of NNV [15,171,250]. It has been suggested that viral replication in these cell-lines and earlier failures in established cell lines may be due to the existence of a specific receptor for NNV [171].

However, in recent years the number of cell lines reported to be susceptible to NNV has increased substantially. Several of these cell lines have been derived from grouper; either from the brain tissue or the snout or the eye. GB cells, already mentioned, were obtained from the brain of yellow grouper (*E. awoara*), GBC1 and GB11 were derived from orange-spotted grouper (*E. coioides*) and *E. moara* brain (EMB) from kelp grouper; ELGSN was obtained from the snout of giant grouper (*E. lanceolatus*) and SIGE from the eye of orange-spotted grouper [104,251,252,253,254]. Other cell cultures derived from the brain of other fish species are: LJB, developed from sea perch (already mentioned), Chinese perch brain (CPB) cells from mandarin fish (*Siniperca chuatsi*), *Trachinotus ovatus* brain (TOGB) cells from golden pompano and DLB-1, also previously mentioned, from European sea bass [232,255,256]. Moreover, OLHNI cells derived from the caudal fin of medaka (*Oryzias latipes*), *Trachinotus ovatus* head kidney (TOHK) and trachinotus ovatus kidney TOK from golden pompano, Japanese flounder skin (JFSK), SISK and SISS from the kidney and spleen, respectively, of Asian sea bass/barramundi [257,258,259,260,261,262] have also been reported to be suitable for NNV propagation. It should be noted that all these susceptibility assays have been performed only with RGNNV strains, probably because this is the predominant genotype in Asian countries, where all these cell lines have been developed.

Other cell lines derived from fish susceptible to NNV have been demonstrated to be persistently infected with the virus. This is the case of BB derived from the brain of Asian sea bass/barramundi and BMGB from brown marbled grouper brain, but they differ in the mechanism supporting viral persistence. Thus, whereas increased Mx expression was observed in BB cells [263], in BMGB persistence was not associated with Mx expression [264].

NNV replication has also been tested in mammalian cell lines. However, the temperature chosen was 28 °C, not 37 °C, for two reasons: this is the optimal temperature for viral replication (20–30 °C) [171] and because the NNV polymerase is not active at 37 °C [171,250]. Studies performed with human cells indicated that HeLa, 293T, and A549 cell lines only supported betanodavirus replication when transfection was performed [265]. However, Cos-1 cells, derived from African green monkey kidney, and DBT, a murine astrocytoma cell line, have been reported to show different degrees of susceptibility to betanodavirus strains. As a result, whereas in Cos-1 cells the titer of virus obtained was lower than that from infected fish cell lines [11], the RGNNV production in DBT cells was 10-fold-higher than in the fish cell line E-11 [266].

### 5.2. Diagnostic Procedures

Several diagnostic methods have been developed since the appearance of the disease. First diagnoses were based on the observation of abnormal swimming behavior and the typical histopathological lesions (vacuolation) in the brain and retina of infected fish [75,103]. However, histopathology was soon considered only appropriate for a presumptive diagnosis and confirmation by immunological methods, indirect fluorescent antibody (IFAT) or by immunoperoxidase staining was recommended [267]. Isolation in cell culture, achieved in 1996 [10], was a big breakthrough in VER diagnosis and for two decades it was considered the reference method to detect NNV infection, followed by immunological or molecular identification [245]. Although in recent years, as described in the previous epigraph, the number of cell lines reported to be susceptible to NNV infection has increased considerably, most isolations are still performed on SNN-1 or E-11 cells. However, this procedure is time-consuming and shows low sensitivity, which leads to false negatives, especially when fish with a low viral load are analyzed. In addition, a successful viral isolation is only achieved when brain tissues are used, making it necessary to kill the fish. Therefore, the use of molecular or serological techniques has gained importance over recent years [245]. 

Several PCR-based techniques (RT-PCR, nested PCR and RT-qPCR) targeted to one or both genomic segments have been reported [16,128,141,268,269,270,271,272,273,274,275,276,277,278]. Nishizawa et al. [268] reported the first RT-PCR protocol, based on the amplification of a 430 bp fragment of the T4 region, capable of detecting an SJNNV strain. This protocol later also proved to be useful for the detection of other NNV genotypes [16] and, as indicated in the epigraph on taxonomic classification, led to the present NNV classification and has been extensively used to type new isolates from different geographic origins [245]. However, some identification problems were observed due to the existence of genetic diversity [277,279] and new protocols were developed targeting more conserved regions of RNA2 or including a second amplification round [141,278,280]. Some of these PCR protocols have been used in non-lethal analyses (using blood, sperm or ovarian tissue) and although it was necessary to use nested PCR, detection levels were similar to those obtained in brain tissue [141,179]. Further developments of the PCR technique have led to a PCR method that allows RGNNV and SJNNV genotyping [273] and to a nanoparticle-based paper lateral flow biosensor (LFB) for visual detection of RT-PCR products in biological samples using gold nanoparticles with a detection limit of 270 pg [281]. Lateral flow paper biosensors have demonstrated to be attractive analytical platform for detection of pathogens because they allow and accurate, rapid and sensitive diagnostics and are also appropriate for field analysis [281].

In recent years RT-PCR detection has given way to RT-qPCR assays and different protocols have been published, targeting one or both genomic segments [128,269,270,271,272,275,276]. In addition, a real time procedure combined with high resolution melting (HRM) has been proposed for NNV detection and genotyping [274]. RT-qPCR has also used for non-lethal detection, testing blood, gills and caudal fin samples [282].

NNV detection has also been accomplished through isothermal amplification methods which are aimed at viral detection with no special equipment [283,284,285,286]. These include nucleic acid sequence-based amplification (NASBA), loop-mediated isothermal amplification (LAMP) and cross priming isothermal amplification coupled with lateral flow dipstick (CPA-LFD). NASBA showed a detection limit between 1.0 and 0.1 TCID_50_ and between 10^3^ and 10^2^ copies of synthetic RNA transcript [283], LAMP showed a sensitivity 100-fold higher than that of nested PCR [284,286] and the detection limit of CPA-LFD was 10 RNA copies/μl comparable to that of RT-qPCR [285].

Serological analysis, mainly an enzyme linked immunosorbent assay (ELISA), has been used for NNV detection since the first disease outbreaks in eggs, larvae and brood stocks [203]. Since then, different ELISA protocols for fish antibodies and viral antigen detection have been reported [207,287,288,289,290,291,292]. 

However, ELISA is problematic for detecting fish antibodies due to its low reproducibility and high background optical density (OD) [293,294], caused by non-specific reactions between antibodies and NNV particles [295]. To solve this problem, a sandwich ELISA using immobilized fish sera has recently been reported [295]. In this ELISA protocol, NNV-specific antibodies could be indirectly detected by detecting NNV antigens captured by fish IgM immobilized onto ELISA plate wells. In addition, antiserum against fish IgM is not required, which means that NNV-specific antibodies are detectable from any fish species using only one antiserum against NNV [295].

The last modification of the ELISA technique reported for NNV detection is an enzyme linked apta-sorbent assay (ELASA) procedure, in which aptamers are used as substitutions of antibodies [296]. Unfortunately, the sensitivity of this procedure cannot be compared with that of the reported ELISA assays, because the detection limit was not provided in the same units.

Finally, in situ hybridization procedures have also been tested for NNV diagnosis, using cDNA-RNA or RNA-RNA probes [185,297]. cDNA-RNA hybridization was used to detect NNV in different organs of experimentally infected European sea bass [185] and RNA-RNA probes have been assayed using the brain tissue of sevenband grouper and also in vitro using SSN-1 cells [295].

Despite all the significant progress achieved in NNV detection over the last few years, recent advances linked to the use of nanotechnology, already reported in human and animal viruses [298,299,300,301,302,303,304,305,306], could provide a substantial improvement in these diagnostics in the near future. Different nanostructures are available at present, such as nanoparticles (NP), carbon nanotubes (CNT), dendrimers, and quantum dots (QDs) among others, and can be applied to the identification of nucleic acids, proteins and viral particles as well as antibodies [307,308]. To the best of our knowledge, nano-based diagnostic methods have only been used twice for the detection of fish viruses—the infectious pancreatic necrosis virus [309] and NNV [281]. The use of a nanoparticle-based LFB for the detection of NNV amplification products enables accurate and fast diagnostics under field conditions, although does not increase sensitivity compared to RT-qPCR assays.

### 5.3. Control of the Disease

Viral diseases are not easy to control once they have been introduced into an aquaculture system and, among them, NNV infections are especially difficult because of the high stability of the NNV particle in the environment [197]. Current control strategies have not been changed substantially with respect to those included in previous reviews [163,245] and rely mainly on good husbandry practices, including biosecurity and sanitation. Briefly, the virus can be completely inactivated by means of chemical disinfectants such as sodium hypochlorite, calcium hypochloride, benzalkonium chloride, chloroquine and iodine [310,311] or by other chemical (ammonium chloride, ozone) or physical treatments (heat, ultra-violet light) [197,311]. The prevention of vertical transmission through the selection of NNV-free breeders is also commonly used. This selection can be performed by testing gonadal fluids, eggs and blood samples with ELISA or PCR-based techniques [128,179,205,206,207]. The vaccination of broodstock has also been reported as a promising method for avoiding vertical transmission [312].

Although at present the use of therapeutic measures is not extended in aquaculture facilities, a number of interesting studies have been performed to search for useful antiviral compounds to control NNV infections [313,314,315,316,317,318,319]. In a recent study, 1000 known drugs, 600 natural products and 400 bioactive components, have been tested for their activity against NNV in a cell viability-based screening assay [314]. This study indicated that proadifen hydrochloride, a cytochrome P 450 inhibitor, showed strong anti-NNV activity. The broad-spectrum antiviral drug Ribavirin has also been demonstrated to inhibit NNV replication, both in vitro and in vivo, in zebra fish larvae used as an experimental model [315,318]. The most recent reports have assessed the anti-NNV activity of chlorpromazine hydrochloride, an inhibitor of clathrin-mediated endocytosis [320] and of isoprinosine, a synthetic purine derivative with immunomodulatory and antiviral properties [321]. Chlorpromazine hydrochloride has shown successful inhibition of NNV infection in SISK cells [319] and isoprinosine was tested both on SSN-1 cells and zebra fish, showing an inhibitory effect of NNV infection. Besides, single-walled carbon nanotubes (SWCNTs) were used for drug delivery to improve anti-NNV activity [321]. Carbon nanotubes, but multiwalled ones, MWCNTs, were also used as a delivery system in a targeted CNS antiviral therapy. In this assay, zebra fish infected with pearl grouper nervous necrosis virus (PGNNV) was exposed to MWCNTs conjugated with polyethylenimine, ribavirin and PGNNV-specific nanobody. Results obtained indicated an obvious accumulation of the nanotubes in the brain of infected fish and a strong anti-PGNNV activity [322].

The use of different peptides, antimicrobial peptides (AMP) and affinity peptides (AFP) has also been explored for controlling NNV infections [313,317,323]. The AMP tested, epinecidin-1 and tilapia hepcidin1–5, have shown in vivo antiviral activity against NNV. Whereas treatment with hepcidin helped to reduce the viral load during infection, Epi-1 cleared the virus during and after the infection [313,323]. The anti-NNV AFPs specifically bound the virus, aggregating or disrupting the viral particles and inhibiting viral infection, by reducing the contact between the virus and cell surface [317].

The screening of potential anti-NNV compounds has also included the use of nucleic acid ligands, called aptamers [316]. These aptamers, with a high degree of affinity and specificity for many targets [324] and already used in viral research [325,326], have been demonstrated to bind to the NNV capsid protein and to reduce fish mortality.

## 6. Prevention

Due to the aforementioned difficulties of controlling VER, special emphasis has been made on vaccine development to prevent the disease. Different vaccine approaches have been looked into in recent years and several have been included in different reviews [163,245]; therefore, we will focus on the most recent ones. It is also worth mentioning that at present, two commercial inactivated (formalin-killed) vaccines against the RGNNV genotype, *Alpha ject micro® 1Noda* (Pharmaq) and Icthiovac*® VNN* (Hipra) are available for sea bass vaccination in the Mediterranean market.

NNV inactivation has been demonstrated to be one of the most effective procedures to achieve a high degree of protection in different species including convict/seven-band grouper [327], orange-spotted grouper [225,312,328], potato grouper (*E. tukula*) [312], brown marbled grouper [329], giant grouper [330], Asian sea bass/barramundi [331] and Atlantic sea bass [289,332]. The inactivation procedures included chemical (formalin, binary ethylenimine, BEI, and β-propiolactone) [225,289,312,327,328,329,331] and physical treatments (heat and UV) [289,332]. Regarding chemical treatments, Kai et al. [328] reported that the use of a BEI- inactivated vaccine resulted in greater protection (relative percent survival, RPS: 79%–95%) than a formalin-inactivated vaccine (RPS: 39%–43%). However, NNV inactivation with formalin was demonstrated to be more effective than inactivation with β-propiolactone [289]. A formalin-inactivated vaccine also provided a high level of protection in brown-marbled grouper (RPS: 86%–100%) [329]. In so far as physical treatments, although heat treatment did not elicit neutralizing antibodies against NNV [289], the UV-inactivated vaccine showed neutralizing activity and an RPS of 57.9% [332].

Another strategy for NNV vaccine development has been the use of subunit vaccines [163]. The latest studies include viral protein expression in recombinant yeast *Sacharomyces cerevissae* [333] or tobacco chloroplasts [334] as well as in *Escherichia coli* [335] and the use of a linear array epitope (LAE) technique [336]. The expression of the viral protein as virus-like particles (VLPs) has also been achieved in the yeast *Yarrowia lipolytica* [337]. The efficacy of recombinant proteins or VLPs has also been assessed in other studies [338,339,340,341,342,343,344] and the stability of the VLP vaccine after lyophilization has been demonstrated [345].

Protection conferred by recombinant-protein vaccines has been reported to be high, i.e., vaccinated giant grouper RPS values above 72% [336] and increasing up to more than 80% in Atlantic sea bass [340]. Recently, total protection has been claimed in vaccinated convict/seven band grouper and European sea bass [335,341].

Finally, the potential use of DNA vaccines to prevent VER outbreaks has been explored in turbot, grouper, European and Asian seabass [346,347,348,349,350]. The protection obtained was moderate in grouper and European sea bass (RPS: 43%–47% and 45%, respectively) [348,349], but considerably higher in Asian sea bass (RPS 77%) [350]. No protection was observed in turbot [346]. Whereas specific anti-NNV antibodies were only detected in grouper and Asian sea bass [347,349], the expression of cell-mediated cytotoxicity-related genes was observed in grouper and European sea bass [348,349], but it was not tested in Asian seabass [347].

All these vaccines have mainly been tested in larvae and juvenile fish, because these developmental stages are the most susceptible to the disease [74,163], but also in broodstock fish and the results obtained indicate that it may be a useful tool for preventing vertical transmission [312,331], as previously indicated.

The vaccine delivery methods tested include bath, intramuscular (i.m.) and intraperitoneal (i.p.) injection and oral vaccination. The bath method has mainly been used for inactivated vaccines, which have also been tested by i.m. or i.p. injection [289,328,329,331,332,349]. Injection has also been used for DNA vaccines [347,349] and subunit vaccines [335,336,338,340,341,342] and oral immunization strategies have been used either for inactivated, subunit or DNA vaccines [225,333,334,335,341,343,348,351,352]. The oral vaccine was given to the fish mixed with food [343] or by oral gavage [333,341] and encapsulated using either chemical compounds [225,348] or Artemia [353]. Furthermore, an oral inactivated vaccine has been demonstrated to confer protection in seven-band grouper after supplementation with capsaicin (a natural substance exerting an intestinal inflammatory reaction) [352].

Finally, a live vaccine has been tested in convict/seven band grouper [354]. Fish were i.m. injected with an NNV strain at 10^4.3^ TCID_50_/ fish at 17 °C, which is not an appropriate temperature for VER development. Although 10.5% mortality was observed in the vaccinated fish, mortality recorded after the challenge with a homologous NNV strain was very low, yielding an RPS value of 95.8%. Attenuated vaccines have yet to be developed for NNV prevention, as reported for other fish viruses such as Cyprinid Herpesvirus 3 or Infectious hematopoietic necrosis virus [355,356]. However, recombinant NNV strains harboring mutations in the capsid protein and in the 3’NCR of RNA2 are promising candidates for attenuated vaccine development [39,177,221].

## 7. Conclusions

NNV infections are currently causing serious problems, mainly in the Mediterranean, Asian and Australian marine aquaculture industry. Over the last 30 years, the body of knowledge about NNV and VER disease has increased considerably; reliable and fast diagnostic techniques have been developed; epidemiological studies have shown the widespread distribution of NNV, especially of the RGNNV genotype, as well as the high number of susceptible and carrier fish species, which is constantly increasing. Additionally, transmission routes have been well demonstrated, including the role of invertebrates and wild fish. Finally, the considerable effort made to research prophylaxes should help to minimize fish farming losses in the short term. However, an increase in the epizootics in presently affected species is likely, as well as a greater risk of disease outbreaks in other farmed species not yet considered susceptible, because many aspects of virus biology and virulence mechanisms are not completely understood and because of global warming. Furthermore, increasing ocean temperatures may favor the number of reported VER episodes in wild species and contribute to the decline of some endangered species.

## Figures and Tables

**Table 1:** Marine fish species affected in natural outbreaks by viral encephalopathy and retinopathy (VER) or viral nervous necrosis (VNN).

Order	Family	Common Name	Latin Name	Status ^a^	Genotype	References	Geographic Range
*Centrarchiformes*	*Latridae*	Striped trumpeteer	*Latris lineata*	F	ND ^b^	[74]	Australia
	*Oplegnathidae*	Japanese parrotfish	*Oplegnathus fasciatus*	F	ND	[75]	Japan
		Spotted knifejaw	*O. punctatus*	F	RGNNV	[76,77]	Japan
		Australian bass	*Macquaria novemaculeata*	F	RGNNV	[46]	Australia
*Gadiformes*	*Gadidade*	Atlantic cod	*Gadus morhua*	F	BFNNV	[34,44,47,77,78]	Canada, USA, Norway, UK
		Pacific cod	*Gadus macrocephalus*	F	BFNNV	[59,79]	Japan, China
		Haddock	*Melanogrammus aeglefinus*	F	BFNNV	[44]	Canada, Atlantic USA
*Gonorynchiformes*	*Chanidae*	Milk fish	*Chanos chanos*	W	ND	[80]	India
*Mugiliformes*	*Mugilidae*	Flathead grey mullet	*Mugil cephalus*	F	RGNNV	[81]	Israel
		Golden mullet	*Liza auratus*	W	ND	[82]	Iran
		Leaping mullet	*L. saliens*	W	ND	[82]	Iran
*Perciformes*	*Centropomatidae*	Asian sea bass/Barramundi	*Lates calcarifer*	F	RGNNV	[48,68,81,83,84,85,86]	Taiwan, India, Singapore, Malaysia, Australia, Israel, China, Indonesia
		Japanese sea bass	*Lateolabrax japonicus*	F	ND	[87]	Japan
	*Ephippidae*	Orbicular batfish	*Platax orbicularis*	F	RGNNV	[88]	French Polynesia
	*Carangidae*	Striped Jack	*Pseudocaranx dentex*	F	SJNNV ^c^	[6]	Japan
		Purplish amberjack	*Seriola dumerili*	F	ND	[76]	Japan
		Pompano	*Trachinotus blochii*	F	RGNNV	[89]	Malaysia
		Permit	*T. falcatus*	F	RGNNV	[83,84]	Taiwan
		Golden pompano	*T. ovatus*	F	RGNNV	[90]	China
	*Lutjanidae*	Firespot snapper	*Lutjanus erythropterus*	F	RGNNV	[83,91]	Taiwan, Malaysia
	*Moronidae*	European sea bass	*Dicentrarchus labrax*	F W	RGNNV, SJ/RG ^d^ RGNNV	[3,4,63,77,81,92,93,94,95] [96]	Martinique, Spain, Portugal, Mediterranean Italy
	*Pomacentridae*	Clownfish,	*Amphiprion sebae Bleeker*	F	RGNNV	[97]	India
	*Rachycentridae*	Cobia	*Rachycentron canadum*	F	RGNNV	[84]	Taiwan
	*Sciaenidae*	Red drum	*Sciaenops ocellatus*	F	RGNNV	[81,98]	Korea, Israel
		Shi drum	*Umbrina cirrosa*	W/F	RGNNV	[77,95,99,100]	France, Italy, Adriatic Sea
		White seabass	*Atractoscion nobilis*	F	RGNNV	[73]	California (USA)
	*Scombridae*	Pacific bluefin tuna	*Thunnus orientalis*	F	RGNNV	[101]	Japan
	*Serranidae*	White grouper	*Epinephelus aeneus*	F	RGNNV	[81]	Israel
		Red spotted grouper	*E. akaara*	F	ND	[102,103]	Taiwan, Japan
		Yellow grouper	*E. awooara*	F	RGNNV	[104]	Taiwan, China
		Duskytail grouper	*E. bleekeri*	F	RGNNV	[69]	China
		Orange-spotted grouper	*E. coioides*	F	RGNNV	[84,105]	Philippines, Taiwan
		Golden grouper	*E. costae*	W	RGNNV	[96,106]	Italy, Algeria
		Blackspotted grouper	*E. fuscogutatus*	F	RGNNV	[85,91,102]	Taiwan, Malaysia
		Dragon/Giant grouper	*E. lanceolatus*	F	RGNNV	[68,107,108]	China, Taiwan, Australia
		Malabar grouper	*E. malabaricus*	F	RGNNV	[107]	Taiwan
		Dusky grouper	*E. marginatus*	W	RGNNV	[96,106,109]	Italy, Algeria, Spain
		Kelp grouper	*E. moara*	F	ND	[110]	Japan
		Sevenband grouper	*E. septemfasciatus*	F	ND	[111,112]	Japan, Korea
		Greasy grouper	*E. tauvina*	F	RGNNV	[15,113,114]	Singapore
		Hybrid grouper	*E. fuscoguttatus x E. lanceolatus*	F	RGNNV	[115]	China, Indonesia
		Humpback grouper	*Chromileptes altivelis*	F	RGNNV	[46,83,84,116,117,118]	Taiwan, Indonesia, Malasia, Vietnam, Australia,
		Spottet coral grouper	*Plectropomus maculatus*	F	ND	[119]	Thailand
	*Siganidae*	Dusky sinefoot	*Siganus fuscescens*	F	RGNNV	[68]	China
	*Sparidae*	Gilthead seabream	*Sparus aurata*	F	RG/SJ ^d^	[55,57,120]	Iberian Peninsula, Italy, Arabian Gulf
		Sobaity seabream	*Sparidentex hasta*	F	ND	[120]	Arabian Gulf
	*Sparidae*	White seabream	*Dioplodus sargus*	F	RGNNV	[95]	Italia, Adriatic sea
*Pleuronectiformes*	*Paralicthyidae*	Japanese flounder	*Paralichthys olivaceus*	F	RGNNV	[16,42,121]	Japan, Korea
	*Pleuronectidae*	Barfin flounder	*Verasper moseri*	F	BFNNV	[42,76]	Japan
		Atlantic halibut	*Hippoglossus hippoglossus*	F	BFNNV	[122,123,124]	Norway, United Kingdom
	*Scophthalmidae*	Turbot	*Scophthalmus maximus*	F	TNNV	[43]	Norway
	*Soleidae*	Dover sole	*Solea solea*	F	BFNNV	[77]	United Kingdom
		Senegalese sole	*S. senegalensis*	F	SJNNV, RG/SJ	[53,55]	Iberian Peninsula
*Scorpaeniformes*	*Sebastidae*	Oblong rockfish	*Sebastes oblongus*	F	RGNNV	[121]	Korea
*Tetraodontiformes*	*Monacanthidae*	Thread-sail filefish	*Stephanolepis cirrhifer*	F	ND	[125]	Thailand
	*Tetraodontidae*	Tiger puffer	*Takifugu rubripes*	F	TPNNV ^c^	[110]	Japan

^a^. F, farmed, W, wild; ^b^, Not determined, ^c^. Based on Nishizawa et al. [42], ^d^. Reassortant RGNNV/SJNNV.

**Table 2:** Detection of nervous necrosis virus (NNV) in asymptomatic farmed fish species.

Order	Family	Common Name	Latin Name	Habitat	Geno-type	Detection	Reference	Geographic Range
*Anguilliformes*	*Muraenidae*	Blue ribbon eel	*Rhinomuraena quaesita*	Marine ^a^	ND ^b^	PCR	[126]	Korea/Indonesia ^d^
*Beryciformes*	*Monocentridae*	Pinecone fish	*Monocentris japonica*	Marine ^a^	ND	PCR	[126]	Korea/Japan ^d^
*Centrarchiformes*	*Oplegnathidae*	Japanese parrotfish	*Oplegnathus fasciatus*	Marine	ND	PCR	[66,127]	Japan, Korea
*Clupeiformes*	*Engraulidae*	Japanese anchovy	*Engraulis japonicus*	Marine ^a^	RGNNV	PCR	[126]	Korea/ Japan^d^
*Gonorynchiformes*	*Chanidae*	Milk fish	*Chanos chanos*	Marine/ freshwater ^a^	ND	PCR	[126]	Korea/Japan ^d^
*Mugiliformes*	*Mugilidae*	Flathead grey mullet	*Mugil cephalus*	Farmed	RGNNV	PCR	[95]	Italy, Adriatic sea
*Perciformes*	*Acanthuridae*	Yellow tang	*Zebrasoma flavescens*	Marine ^a^	ND	PCR	[126]	Korea/Singapore ^d^
	*Carangidae*	Look down fish	*Selene vomer*	Marine ^a^	ND	PCR	[126]	Korea/N. America ^d^
		Golden pompano	*Trachinotus ovatus*	Marine	RGNNV	PCR	[68]	China
	*Lateolabracidae*	Chinese seabass	*Lateolabrax sp*	Marine	RGNNV	PCR	[66]	Japan
		Japanese sea perch	*Lateolabrax japonicus*	Marine	RGNNV	PCR	[68]	China
	*Moronidae*	European sea bass	*Dicentrarchus labrax*	Marine	RGNNV	PCR, CC ^c^,	[128]	Italy
	*Mullidae*	Red mullet	*Mullus barbatus barbatus*	Marine	RGNNV	PCR, CC	[95]	Italy, Adriatic Sea
	*Polycentridae*	South American leaf fish	*Monocirrhus polyacanthus*	Freshwater ^a^	RGNNV	PCR, CC	[126]	Korea/Amazon ^d^
	*Pomacentridae*	Three spot damsel	*Dascyllus trimaculatus*	Marine ^a^	RGNNV	PCR, CC	[126]	Korea/Singapore ^d^
	*Serranidae*	Brown-spotted grouper	*E. chlorostigma*	Marine	ND	PCR	[120]	Arabian Gulf
		Giant grouper	*E. lanceolatus*	Marine	RGNNV	PCR	[68]	China
								
		Redspot grouper	*Epinephelus akaara*	Marine	RGNNV	PCR	[66]	Japan
		Yellow grouper	*E. awooara*	Marine	RGNNV	PCR	[68]	China
	*Serrasalmidae*	Red piranha	*Pygocentrus nattereri*	Freshwater ^a^	RGNNV	PCR, CC	[126]	Korea/Amazon ^d^
	*Siganidae*	Dusky sinefoot	*Siganus fuscescens*	Marine	RGNNV	PCR	[68]	China
	*Sparidae*	Gilthead seabream	*Sparus aurata*	Marine	ND, RGNNV	PCR	[63,95,129,130]	France (Atlantic),Mediterranean
		Red seabream	*Pagrus major*	Marine	RGNNV	PCR	[66,131]	Japan, Korea
	*Stromateidae*	Silver pomfret	*Pampus argenteus*	Marine	RGNNV	PCR	[68]	China
*Pleuronectiformes*	*Paralicthyidae*	Japanese flounder	*Paralichthys olivaceus*	Marine	RGNNV	PCR	[66,127]	Japan, Korea
	*Pleuronectidae*	Marbled sole	*Pleuronectes yokohamae*	Marine	RGNNV	PCR	[66]	Japan
		Stone flounder	*Kareius bicoloratus*	Marine	RGNNV	PCR	[131]	Korea
	*Sciaenidae*	Red drum	*Sciaenops ocellatus*	Marine	RGNNV	PCR	[68]	China
	*Scopththalmidae*	Turbot	*Scophthalmus maximus*	Marine	RGNNV	PCR, CC	[132]	Spain
*Scorpaeniformes*	*Sebastidae*	Black rockfish	*Sebastes inermis*	Marine	RGNNV	PCR	[66]	Japan
*Scorpaeniformes*		Oblong rockfish	*S. oblongus*	Marine	RGNNV	PCR	[66]	Japan
		Schlegel’s black rockfish	*S. schlegelii*	Marine	RGNNV	PCR	[131]	Korea
	*Sebastidae*	Spotbelly rockfish	*S. pachycephalus*	Marine	RGNNV	PCR	[66]	Japan
	*Synanceiidae*	Devil stinger	*Inimicus japonicus*	Marine	RGNNV	PCR	[66]	Japan
*Tetraodontiformes*	*Monacanthidae*	Black scraper	*Thamnaconus modestus*	Marine	ND	PCR	[66]	Japan
	*Tetraodontidae*	Tiger puffer	*Takifugu rubripes*	Marine	RGNNV	PCR	[66]	Japan
*Syngnathiformes*	*Centriscidae*	Shrimp fish	*Aeoliscus strigatus*	Marine ^a^	ND	PCR	[126]	Korea/Japan ^c^

^a^, ornamental fish; ^b^, Not determined; ^c^, isolation in cell culture; ^d^, place of detection/fish procedence.

**Table 3:** Freshwater fish species affected –in natural outbreaks- by viral encephalopathy and retinopathy (VER).

Order	Family	Common Name	Latin Name	Status	Genotype	Reference	Geographic Range
*Acipenseriformes*	*Acipenseridae*	Danube sturgeon	*Acipenser gueldenstaedtii*	Farmed	ND ^a^	[133]	Greece
*Anguilliformes*	*Anguillidae*	European eel	*Anguilla anguilla*	Farmed	RGNNV	[83,84]	Taiwan
*Cypriniformes*	*Cyprinidae*	Goldfish	*Carassius auratus*	Ornamental	ND	[134]	India
		Rainbow sharkminnow	*Epalzeorhynchos frenatum*	Ornamental	ND	[134]	India
*Cyprinodontiformes*	*Poeciliidae*	Guppy	*Poecilia reticulata*	Farmed	RGNNV	[17]	Singapore
*Perciformes*	*Blenniidae*	Freshwater blenny	*Salaria flubiatilis*	Wild	RGNNV	[135]	Spain
	*Centrarchidae*	Largemouth black bass	*Micropterus salmoides*	Farmed	RGNNV	[136]	Italy
	*Cichlidae*	Tilapia	*Oreochromis niloticus*	Farmed	RGNNV	[49,137]	Europe, Indonesia
	*Eleotridae*	Sleepy cod	*Oxyeleotris lineolatus*	Farmed	RGNNV	[46]	Australia
	*Moronidae*	Hybrid striped bass	*Morone saxatilis* x *M. chrysops*	Farmed	RGNNV	[136]	Italy
	*Percidae*	Pike-perch	*Sander lucioperca*	Farmed	RGNNV	[136]	Italy
*Siluriformes*	*Plotosidae*	Catfish	*Tandanus tandanus*	Farmed	ND	[74]	Australia
	*Siluridae*	Chinese catfish	*Parasilurus asotus*	Farmed	RGNNV	[84]	Taiwan

^a^, Not determined.

**Table 4:** Detection of nervous necrosis virus (NNV) in asymptomatic wild fish species.

Order	Family	Common Name	Latin Name	Geno- type	Detection	Reference	Geographic Range
*Anguilliformes*	*Anguillidae*	European eel	*Anguilla anguilla*	SJNNV	PCR	[138]	Spain
	*Congridae*	Conger eel	*Rhynchocymba nystromi*	RGNNV	PCR	[68]	China
*Atheriniformes*	*Atherinidae*	Mediterranean sand smelt	*Atherina hepsetus*	RGNNV	qPCR ^a^	[58]	Greece
		Ring-tailed cardinalfish	*A. aureus*	RGNNV	PCR	[68]	China
*Aulopiformes*	*Synodontidae*	Threadfin saury	*Saurida filamentosa*	RGNNV	PCR	[68]	China
		Brushtooth lizardfish	*S. lessepsianus*	RGNNV	PCR	[130]	Mediterranean
		Snakefish	*Trachinocephalus myops*	RGNNV	PCR	[68]	China
*Bathrachoidiformes*	*Bathrachoididae*	Lusitania toadfish	*Halobatrachus didactylus*	RGNNV	PCR, CC ^b^	[64]	Spain
*Beloniformes*	*Belonidae*	Garpike	*Belone belone*	RGNNV	PCR, CC	[62]	Italy
	*Exocoetidae*	Mediterranean flyingfish	*Cheilopogon heterurus*	RGNNV	PCR	[58]	Greece
*Centrarchiformes*	*Latridae*	Striped trumpeter	*Latris lineata*	RGNNV	PCR	[46]	Australia
	*Oplegnathidae*	Japanese parrotfish	*Oplegnathus fasciatus*	RGNNV	PCR	[66]	Japan
*Clupeiformes*	*Clupeidae*	Pilchard	*Sardina pilchardus*	RGNNV	PCR, CC	[58,62,65]	Italy. Tunisia
		Round sardinella	*Sardinella aurita*	RGNNV	PCR	[65,130]	Mediterranean
		Sardine	*S. jussieui*	RGNNV	PCR	[68]	China
	*Dussumieriidae*	Slender rainbow sardine	*Dussumieria elopsoides*	RGNNV	PCR	[130]	Mediterranean
	*Engraulidae*	Commerson´s anchovy	*Tolephorus commersonnii*	RGNNV	PCR	[68]	China
		European anchovy	*Engraulis encrasicolus*	RGNNV	PCR	[58]	Greece
*Gadiformes*	*Gadidae*	Atlantic cod	*Gadus morhua*	BFNNV	PCR	[47]	Norway
		Poor cod	*Trisopterus minutus*	RGNNV	PCR, qPCR	[56,58]	Italy, Greece
		Pollock	*Pollachius pollachius*	ND	PCR	[47]	Norway
		Saithe	*P. virens*	ND	PCR	[47]	Norway
		Whiting	*Merlangius merlangus*	RGNNV	PCR	[62]	Italy
	*Merluciidae*	European hake	*Merlucius merlucius*	RGNNV	PCR, CC	[58,62]	Italy, Greece
	*Macrouridae*	Glasshead grenadier	*Hymenocephalus italicus*	ND	PCR	[139]	Italy
*Heterodontiformes*	*Heterodontidae*	Japanese bullhead shark	*Heterodontus japonicus*	RGNNV	PCR	[66]	Japan
*Kurtiformes*	*Apogonidae*	Red stoplight cardinalfish	*Apogon erythrinus*	RGNNV	PCR	[68]	China
*Mugiliformes*	*Mugilidae*	Flathead grey mullet	*Mugil cephalus*	RGNNV	PCR, CC	[58,62,66,69]	Japan, Italy, China, Greece
		Golden mullet	*Liza auratus*	RGNNV	PCR	[65]	Tunisia
		Thicklip grey mullet	*Chelon labrosus*	RGNNV, SJNNV	PCR	[64]	Spain
*Notacanthiformes*	*Notacanthidae*	Shorfin spiny eel	*Notacanthus bonaparte*	ND	PCR	[139]	Italy
*Perciformes*	*Carangidae*	Atlantic horse mackerel	*Trachurus thrachurus*	RGNNV	PCR, CC	[58,62,65]	Italy, Tunisia
		Japanese jack mackerel	*T. japonicus*	RGNNV, SJNNV	PCR, CC	[66,67,68,70,71]	Japan, China
		Mediterranean horse mackerel	*T. mediterraneus*	RGNNV, RG/SJ	PCR, CC	[58,62]	Italy, Greece
		Blue Jack mackerel	*T. picturatus*	ND	PCR, CC	[128]	Italy
		Bigeye trevalli	*Caranx oshimai*	RGNNV	PCR	[69]	China
		Greater amberjack	*Seriola dumerili*	RGNNV, SJNNV	PCR	[58,67]	Japan, Greece
		Japanese amberjack	*S. quinqueradiata*	SJNNV	PCR	[67]	Japan
		Indian threadfish	*Alectis indica*	RGNNV	PCR	[69]	China
		Japanese scad	*Decapterus maruadsi*	RGNNV	PCR	[66]	Japan
		Shrimp scad	*Caranx djedaba*	RGNNV	PCR	[69]	China
		Yellowstripe scad	*Selaroides leptolepis*	RGNNV	PCR	[68,69]	China
		Whitefin trevally	*Carangoides equula*	RGNNV, SJNNV	nPCR ^c^	[67]	Japan
	*Centrolophidae*	Pacific rudderfish	*Psenopsis anomala*	RGNNV	PCR	[68]	China
	*Centropomatidae*	Asian sea bass/ Barramundi	*Lates calcarifer*	RGNNV	PCR	[48]	India
	*Epigonidae*	Cardinal fish	*Epigonus telescopus*	ND	PCR	[139]	Italy
	*Gobiidae*	Black goby	*Gobius niger*	RGNNV	PCR, CC	[62]	Italy
	*Haemulidae*	Trout sweetlips	*Plectorhynchus pictus*	RGNNV	PCR	[69]	China
	*Kyphosidae*	Stripey fish	*Microcanthus strigatus*	RGNNV	PCR	[66]	Japan
	*Labridae*	Ballan wrasse	*Labrus bergylta*	BFNNV, RGNNV	PCR	[50]	Sweden
		Corkwing wrasse	*Symphodus melops*	BFNNV, RGNNV	PCR	[50]	Norway
		Goldsinny wrasse	*Ctenolabrus rupestris*	BFNNV	PCR	[50]	Norway, Sweden
	*Leiognathidae*	Pugnose ponyfish	*Leigognathus insidiator*	RGNNV	PCR	[69]	China
		Berber ponyfish	*L. berbis*	RGNNV	PCR	[69]	China
		Slender ponyfish	*L. elongatus*	RGNNV	PCR	[68]	China
	*Lutjanidae*	Dory snapper	*Lutjanus fulviflamma*	RGNNV	PCR	[69]	China
		Humphead snapper	*L. sanguineus*	RGNNV	PCR	[69]	China
		John’s snapper	*L. johni*	RGNNV	PCR	[69]	China
		Mangrove red snapper	*L. argentimaculatus*	RGNNV	PCR	[69]	China
		Russell’s snapper	*L. russelli*	RGNNV	PCR	[69]	China
	*Moronidae*	European sea bass	*Dicentrarchus labrax*	ND	qPCR	[128]	Italy
	*Mullidae*	Red mullet	*Mullus barbatus barbatus*	RGNNV	PCR, CC	[62,64,130]	Mediterranean
		Surmullet	*M. surmuletus*	RGNNV	PCR, qPCR	[56,58,130]	Mediterranean
		Japanese goatfish	*Upeneus japonicus*	RGNNV	PCR	[68]	China
		Goldband goatfish	*U. moluccensis*	RGNNV	PCR	[130]	Mediterranean
	*Nemipteridae*	Japanese threadfin bream	*Nemipterus japonicus*	RGNNV	PCR	[68]	China
		Randall’s threadfin bream	*N. randalli*	RGNNV	PCR	[130]	Mediterranean
	*Percophidae*	Ray finned fish	*Chrionema chlorotaenia*	RGNNV	PCR	[68]	China
	*Pomacentridae*	Heavenly damselfish	*Pomacentrus coelestis*	RGNNV	PCR	[66]	Japan
	*Priacanthidae*	Red bigeye	*Priacanthus macracanthus*	RGNNV	PCR	[68]	China
	*Sciaenidae*	Goatee croaker	*Umbrina russelli*	RGNNV	PCR	[69]	China
		Big head penah croaker	*Argyrosomus macrocephalus*	RGNNV	PCR	[68,69]	China
		Silver croaker	*A. argentatus*	RGNNV	PCR	[69]	China
		Meagre	*A. regius*	RGNNV, SJNNV	PCR	[140]	Spain
		Brown meagre	*Sciaena umbra*	RGNNV	PCR, qPCR	[58,141]	Italy, Greece
		Hoki	*Johnius belengerii*	RGNNV	PCR	[68]	China
		Shy drum	*Umbrina cirrosa*	RGNNV	PCR	[43]	Italy
	*Scombridae*	Atlantic mackerel	*Scomber scombrus*	ND	PCR	[47,58]	Norway, Greece
		Chub mackerel	*S. japonicus*	RGNNV, SJNNV	PCR	[67]	Japan
		Atlantic bluefin tuna	*Thunnus thynnus*	RGNNV	PCR, qPCR	[58,142]	Japan, Greece
	*Serranidae*	Areolate grouper	*Epinephelus. areolatus*	RGNNV	PCR	[69]	China
		Longfin grouper	*E. megachir*	RGNNV	PCR	[69]	China
		Longspine grouper	*E. fario*	RGNNV	PCR	[69]	China
		Dotted grouper	*E. epistictus*	RGNNV	PCR	[69]	China
		Honeycumb grouper	*E. merra*	RGNNV	PCR	[69]	China
		Kelp grouper	*E. moara*	RGNNV	PCR	[69]	China
		Rock grouper	*E. fasciatomaculatus*	RGNNV	PCR	[69]	China
	*Siganidae*	White-spotted spinefoot	*Siganus oramin*	RGNNV	PCR	[69]	China
	*Sparidae*	Bogue	*Boops boops*	RGNNV	qPCR	[58,128]	Italy, Greece
		Axillary seabream	*Pagellus acarne*	RGNNV	PCR, CC	[64]	Spain
		Black seabream	*Spondyliosoma cantharus*	RGNNV	PCR, CC	[64]	Spain
		Common two-banded seabream	*Diploidus vulgaris*	RGNNV	PCR, CC	[64]	Spain
		Sharpsnout seabream	*D. puntazzo*	RGNNV	PCR, qPCR	[58]	Greece
		Annular seabream	*D. annularis*	ND	qPCR	[198]	Italy
		Red porgy	*Pagrus pagrus*	RGNNV	PCR, qPCR	[58,143]	Spain, Greece
		Salema	*Sarpa salpa*	RGNNV	qPCR	[58]	Greece
		Picarel	*Spicara smaris*	RGNNV	qPCR	[58]	Greece
		Blotched picarel	*S. maena*	RGNNV	qPCR	[58]	Greece
		Gilthead seabream	*Sparus aurata*	RGNNV	PCR, qPCR	[58,95]	Italy, Greece
		Striped seabream	*Lithognathus mormyrus*	RGNNV	PCR	[130]	Mediterranean
	*Sphyraenidae*	European barracuda	*Sphiraena sphiraena*	RGNNV	qPCR, CC	[58,128]	Italy, Greece
	*Terapontidae*	Fourlined terapon	*Pelates quadrilineatus*	RGNNV	PCR	[69]	China
		Trumpeter perch	*P. quadrilineatus*	RGNNV	PCR	[68]	China
		Jarbua terapon	*Terapon jarbua*	RGNNV	PCR	[69]	China
	*Zanclidae*	Moorish idol	*Zanclus cornutus*	RGNNV	PCR	[66]	Japan
*Pleuronectiformes*	*Citharidae*	Branched ray flounder	*Citharoides macrolepidotus*	RGNNV	PCR	[68]	China
	*Pleuronectidae*	European plaice	*Pleuronectes platessa*	ND	PCR	[47]	Norway
		Winter flounder	*Pseudopleuronectes americanus*	BFNNV	PCR, CC	[61]	Canada
	*Citharidae*	Branched ray flounder	*Citharoides macrolepidotus*	RGNNV	PCR	[68]	China
*Siluriformes*	*Plotosidae*	Striped catfish eel	*Plotosus lineatus*	RGNNV	PCR	[66]	Japan
*Scorpaeniformes*	*Scorpaenidae*	Luna lionfish	*Pterois lunulata*	RGNNV	PCR	[66]	Japan
		Red scorpionfish	*Scorpaena scrofa*	RGNNV	qPCR	[58]	Greece
	*Sebastidae*	Black rockfish	*Sebastes inermis*	ND	PCR	[66]	Japan
		Schlegel’s black rockfish	*S. schlegelii*	RGNNV	PCR	[66]	Japan
		Marbled rockfish	*Sebastiscus marmoratus*	RGNNV	PCR	[66]	Japan
	*Triglidae*	Gurnard	*Tigla lyra*	RGNNV	PCR	[62]	Italy
		Tub gurnard	*Chelidonichtys lucerna*	RGNNV	PCR, CC	[64]	Spain
	*Triglidae*	Spiny gurnard	*Lepidotrigla dieuzeidei*	RGNNV	qPCR	[58]	Greece
*Syngnathiformes*	*Fistuliridae*	Red cornetfish	*Fistularia villosa*	RGNNV	PCR	[68]	China
*Trachichthyiformes*	*Trachichthyidae*	Mediterranean slimehead	*Hoplostethus mediterraneus*	ND	PCR	[139]	Italy
*Tetraodontiformes*	*Diodontidae*	Freckled porcupine fish	*Diodon holocanthus*	RGNNV	PCR	[66]	Japan
	*Monacanthidae*	Threadsail filefish	*Stephanolepis cirrhifer*	RGNNV	PCR	[66]	Japan
		Black scraper	*Thamnaconus modestus*	RGNNV	PCR	[66]	Japan
	*Tetraodontidae*	Panther puffer	*Takifugu pardalis*	RGNNV	PCR	[66]	Japan

^a^, real time-PCR; ^b^, isolation in cell culture; ^c^, nested-PCR.

**Table 5:** Detection of nervous necrosis virus (NNV) in shellfish.

Order	Family	Common Name	Latin Name	Genotype	Detection	References	Geografic Range
*Arcida*	*Arcidae*	Granular ark	*Tegillarca granosa*	RGNNV, BFNNV	PCR	[131]	Korea, Japan
*Mytiloida*	*Mytilidae*	Mussel	*Mytilus edulis*	RGNNV, BFNNV	PCR	[131]	Korea
		Mediterranean mussel	*M. galloprovincialis*	RGNNV	PCR	[58,200,201]	Korea, Italy, Greece
*Oegopsida*	*Ommastrephidae*	Japanese common squid	*Todarodes pacificus*	RGNNV	PCR, CC ^a^	[201]	Japan
*Ostreoida*	*Ostreidae*	European flat oyster	*Ostrea edulis*	RGNNV	qPCR ^b^	[58]	Greece
		Pacific oyster	*Cassostrea gigas*	RGNNV, BFNNV	PCR	[131,200]	Korea, France
*Octopoda*	*Octopodidae*	Octopus	*Octopus vulgaris*	ND ^c^	PCR, CC	[202]	Italy
*Decapoda*	*Palinuridae*	Spiny lobster	*Pamulirus versicolor*	ND	PCR	[126]	Japan
	*Pandalidae*	Southern humpback shrimp	*Pandalus hypsinotus*	RGNNV	PCR	[201]	Korea
	*Penaeidae*	Kuruma prawn	*Marsupenaeus japonicus*	RGNNV	PCR	[130]	Mediterranean
	*Portunidae*	Charybdid crab	*Charybdis bimaculata)*	RGNNV	PCR	[201]	Korea
		Blue crab	*Portunus pelagicus*	RGNNV	PCR	[130]	Mediterranean
*Pectinida*	*Pectinidae*	Scallop	*Patinopecten yessoensis*	RGNNV, BFNNV	PCR	[131]	China, Japan
*Veneroida*	*Veneridae*	Clam	*Ruditapes philipinarum*	RGNNV	PCR	[200]	Italy
		Common orient clam	*Meretrix lusoria*	RGNNV, BFNNV	PCR	[131]	Korea
		Chinese cyclina	*Cyclina sinensis*	BFNNV	PCR	[131]	China
		Manila clam	*Venerupis philippinarum*	RGNNV, BFNNV	PCR	[131]	Korea, China, Japan
		Venus clam	*Mercenaria mercenaria*	BFNNV	PCR	[131]	China
		Wrinkled venus clam	*Callista brevisiphonata*	BFNNV	PCR	[131]	China
		Warty venus	*Venus verrucosa*	RGNNV	qPCR	[58]	Greece
*Neogastropoda*	*Muricidae*	Red-mouthed rockshell	*Stramonita haemastoma*	RGNNV	qPCR	[58]	Greece

^a^, isolation in cell culture; ^b^, real time-PCR; ^c^, Not determined.

## References

[1] Glazebrook J.S., Campbell R.S.F., Coppland J.W., Grey D.L. (1987). Diseases of barramundi (Lates calcarifer) in Australia: A review. Management of Wild and Cultured Sea Bass/Barramundi (Lates calcarifer), Proceedings of an international workshopm Darwin, N.T. Australia.

[2] Callinan R.B., Bryden D.I. (1988). Diseases of Australian native fishes. Fish Diseases.

[3] Bellance R., Gallet de Saint-Aurin D. (1998). L’encephalite virale de loup de mer. Caraibes Med..

[4] Breuil G., Bonami J.R., Pepin J.F., Pichot Y. (1991). Viral infection (picorna-like virus) associated with mass mortalities in hatchery-reared sea-bass (*Dicentrarchus labrax*) larvae and juveniles. Aquaculture.

[5] Glazebrook J.S., Heasman M.P., Beer S.W. (1990). Picorna-like viral particles associated with mass mortalities in larval barramundi, *Lates calcarifer* Bloch. J. Fish Dis..

[6] Mori K.-I., Nakai T., Muroga K., Arimoto M., Mushiake K., Furusawa I. (1992). Properties of a new virus belonging to nodaviridae found in larval striped jack (*Pseudocaranx dentex*) with nervous necrosis. Virology.

[7] Comps M., Pépin J.F., Bonami J.R. (1994). Purification and characterization of two fish encephalitis viruses (FEV) infecting *Lates calcarifer* and *Dicentrarchus labrax*. Aquaculture.

[8] Ball L., Hendry D., Johnson J., Ruechert R., Scotti P., Van Regenmortel M.H., Fauquet C.M., Bishop D.H.L., Cartens E.B., Estes M.K., Lemon S.M., Maniloff J., Mayo M.A., McGeoch D.J., Pringle C.R. (2000). Family Nodaviridae. Virus Taxonomy. Seventh Report of the International Committee on Taxonomy of Viruses.

[9] Schneemann A., Ball L.A., Delsert C., Johnson J.E., Nishizawa T., Fauquet C.M., Mayo M.A., Maniloff J., Desselberger U., Ball L.A. (2005). Nodaviridae. Virus Taxonomy, Eighth Report of the International Committee on Taxonomy of Viruses.

[10] Frerichs G.N., Rodger H.D., Peric Z. (1996). Cell culture isolation of piscine neuropathy nodavirus from juvenile sea bass, *Dicentrarchus labrax*. J. Gen. Virol..

[11] Delsert C., Morin N., Comps M. (1997). A fish encephalitis virus that differs from other nodaviruses by its capsid protein processing. Arch. Virol..

[12] Nagai T., Nishizawa T. (1999). Sequence of the non-structural protein gene encoded by RNA1 of striped jack nervous necrosis virus. J. Gen. Virol..

[13] Iwamoto T., Mise K., Takeda A., Okinaka Y., Mori K.I., Arimoto M., Okuno T., Nakai T. (2005). Characterization of striped jack nervous necrosis virus subgenomic RNA3 and biological activities of its encoded protein B2. J. Gen. Virol..

[14] Sommerset I., Nerland A. (2004). Complete sequence of RNA1 and subgenomic RNA3 of Atlantic halibut nodavirus (AHNV). Dis. Aquat. Organ..

[15] Tan C., Huang B., Chang S.F., Ngoh G.H., Munday B., Chen S.C., Kwang J. (2001). Determination of the complete nucleotide sequences of RNA1 and RNA2 from greasy grouper (*Epinephelus tauvina*) nervous necrosis virus, Singapore strain. J. Gen. Virol..

[16] Nishizawa T., Mori K., Furuhashi M., Nakai T., Furusawa I., Muroga K. (1995). Comparison of the coat protein genes of five fish nodaviruses, the causative agents of viral nervous necrosis in marine fish. J. Gen. Virol..

[17] Hegde A., Teh H.C., Lam T.J., Sin Y.M. (2003). Nodavirus infection in freshwater ornamental fish, guppy, *Poicelia reticulata*—Comparative characterization and pathogenicity studies. Arch. Virol..

[18] Venter P.A., Schneemann A. (2008). Recent insights into the biology and biomedical applications of Flock House virus. Cell. Mol. Life Sci..

[19] Krondiris J.V., Sideris D.C. (2002). Intramolecular disulfide bonding is essential for betanodavirus coat protein conformation. J. Gen. Virol..

[20] Wang C.-H., Hsu C.-H., Wu Y.-M., Luo Y.-C., Tu M.-H., Chang W., Cheng R.H., Lin C.-S. (2010). Roles of cysteines Cys115 and Cys201 in the assembly and thermostability of grouper betanodavirus particles. Virus Genes.

[21] Chen N.C., Yoshimura M., Guan H.H., Wang T.Y., Misumi Y., Lin C.C., Chuankhayan P., Nakagawa A., Chan S.I., Tsukihara T. (2015). Crystal Structures of a Piscine Betanodavirus: Mechanisms of Capsid Assembly and Viral Infection. PLoS Pathog..

[22] Wu Y.M., Hsu C.H., Wang C.H., Liu W., Chang W.H., Lin C.S. (2008). Role of the DxxDxD motif in the assembly and stability of betanodavirus particles. Arch. Virol..

[23] Guo Y.X., Dallmann K., Kwang J. (2003). Identification of nucleolus localization signal of betanodavirus GGNNV protein α. Virology.

[24] Guo Y.X., Wei T., Dallmann K., Kwang J. (2003). Induction of caspase-dependent apoptosis by betanodaviruses GGNNV and demonstration of protein α as an apoptosis inducer. Virology.

[25] Wu H.-C.C., Chiu C.-S.S., Wu J.-L.L., Gong H.-Y.Y., Chen M.-C.C., Lu M.-W.W., Hong J.-R.R. (2008). Zebrafish anti-apoptotic protein zfBcl-xL can block betanodavirus protein α-induced mitochondria-mediated secondary necrosis cell death. Fish Shellfish Immunol..

[26] Okinaka Y., Nakai T. (2008). Comparisons among the complete genomes of four betanodavirus genotypes. Dis. Aquat. Organ..

[27] Chen L.J., Su Y.C., Hong J.R. (2009). Betanodavirus non-structural protein B1: A novel anti-necrotic death factor that modulates cell death in early replication cycle in fish cells. Virology.

[28] Su Y.-C., Reshi L., Chen L.-J., Li W.-H., Chiu H.-W., Hong J.-R. (2018). Nuclear targeting of the betanodavirus B1 protein via two arginine-rich domains induces G1/S cell cycle arrest mediated by upregulation of p53/p21. Sci. Rep..

[29] Fenner B.J., Thiagarajan R., Chua H.K., Kwang J. (2006). Betanodavirus B2 is an RNA interference antagonist that facilitates intracellular viral RNA accumulation. J. Virol..

[30] Chen S.P., Wu J.L., Su Y.C., Hong J.R. (2007). Anti-Bcl-2 family members, zfBcl-xL and zfMcl-1a, prevent cytochrome c release from cells undergoing betanodavirus-induced secondary necrotic cell death. Apoptosis.

[31] Su Y.C., Wu J.L., Hong J.R. (2009). Betanodavirus non-structural protein B2: A novel necrotic death factor that induces mitochondria-mediated cell death in fish cells. Virology.

[32] Su Y.C., Hong J.R. (2010). Betanodavirus B2 causes ATP depletion-induced cell death via mitochondrial targeting and complex II inhibition in vitro and in vivo. J. Biol. Chem..

[33] Guo Y.X., Chan S.-W., Kwang J. (2004). Membrane association of greasy grouper nervous necrosis virus protein A and characterization of its mitochondrial localization targeting signal. J. Virol..

[34] Mézeth K.B., Nylund S., Henriksen H., Patel S., Nerland A.H., Szilvay A.M. (2007). RNA-dependent RNA polymerase from Atlantic Halibut Nodavirus contains two signals for localization to the mitochondria. Virus Res..

[35] Panzarin V., Cappellozza E., Mancin M., Milani A., Toffan A., Terregino C., Cattoli G. (2014). In vitro study of the replication capacity of the RGNNV and the SJNNV betanodavirus genotypes and their natural reassortants in response to temperature. Vet. Res..

[36] Souto S., Salgado L.V., Olveira J.G., Bandín I. (2019). Amino acidic substitutions in the polymerase N-terminal region of a reassortant betanodavirus strain causing poor adaptation to temperature increase. Vet. Res..

[37] Rosskopf J.J., Upton J.H., Rodarte L., Romero T.A., Leung M.-Y., Taufer M., Johnson K.L. (2010). A 3’ terminal stem–loop structure in Nodamura virus RNA2 forms an essential cis-acting signal for RNA replication. Virus Res..

[38] Taufer M., Leung M.-Y., Solorio T., Licon A., Mireles D., Araiza R., Johnson K.L. (2008). RNAVLab: A virtual laboratory for studying RNA secondary structures based on grid computing technology. Parallel Comput..

[39] Souto S., Olveira J.G., Dopazo C.P., Borrego J.J., Bandín I. (2018). Modification of betanodavirus virulence by substitutions in the 3’ terminal region of RNA2. J. Gen. Virol..

[40] Kim J.-O.O., Kim W.-S.S., Oh M.-J.J. (2019). Investigation of nervous necrosis virus (NNV) replication in vitro using RNA in situ hybridization. Virus Res..

[41] Fenner B.J., Goh W., Kwang J. (2006). Sequestration and Protection of Double-Stranded RNA by the Betanodavirus B2 Protein. J. Virol..

[42] Nishizawa T., Furuhashi M., Nagai T., Nakai T., Muroga K. (1997). Genomic classification of fish nodaviruses by molecular phylogenetic analysis of the coat protein gene. Appl. Environ. Microbiol..

[43] Johansen R., Sommerset I., Torud B., Korsnes K., Hjortaas M.J., Nilsen F., Nerland A.H., Dannevig B.H. (2004). Characterization of nodavirus and viral encephalopathy and retinopathy in farmed turbot, *Scophthalmus maximus* (L.). J. Fish Dis..

[44] Gagné N., Johnson S.C., Cook-Versloot M., MacKinnon A.M., Olivier G. (2004). Molecular detection and characterization of nodavirus in several marine fish species from the northeastern Atlantic. Dis. Aquat. Organ..

[45] Kim Y.C., Kwon W.J., Min J.G., Kim K., Jeong H.D. (2019). Complete genome sequence and pathogenic analysis of a new betanodavirus isolated from shellfish. J. Fish Dis..

[46] Moody N.J.G., Horwood P.F., Reynolds A., Mahony T.J., Anderson I.G., Oakey H.J. (2009). Phylogenetic analysis of betanodavirus isolates from Australian finfish. Dis. Aquat. Organ..

[47] Nylund A., Karlsbakk E., Nylund S., Isaksen T.E., Karlsen M., Korsnes K., Handeland S., Martinsen R., Mork Pedersen T., Ottem K.F. (2008). New betanodaviruses detected in wild and farmed cod (*Gadus morhua*) in Norway. Arch. Virol..

[48] Binesh C.P., Greeshma C. (2013). Genomic classification of betanodavirus by molecular phylogenetic analysis of the coat protein gene. Arch. Virol..

[49] Keawcharoen J., Techangamsuwan S., Ponpornpisit A., Lombardini E.D., Patchimasiri T., Pirarat N. (2015). Genetic characterization of a betanodavirus isolated from a clinical disease outbreak in farm-raised tilapia *Oreochromis niloticus* (L.) in Thailand. J. Fish Dis..

[50] Korsnes K., Karlsbakk E., Skår C.K., Sælemyr L., Nylund A., Kvamme B.O., Mortensen S. (2017). High nervous necrosis virus (NNV) diversity in wild wrasse (*Labridae*) in Norway and Sweden. Dis. Aquat. Organ..

[51] NaveenKumar S., Shekar M., Karunasagar I., Karunasagar I. (2013). Genetic analysis of RNA1 and RNA2 of Macrobrachium rosenbergii nodavirus (MrNV) isolated from India. Virus Res..

[52] Ho K.L., Gabrielsen M., Beh P.L., Kueh C.L., Thong Q.X., Streetley J., Tan W.S., Bhella D. (2018). Structure of the Macrobrachium rosenbergii nodavirus: A new genus within the Nodaviridae?. PLoS Biol..

[53] Thiéry R., Cozien J., de Boisséson C., Kerbart-Boscher S., Névarez L. (2004). Genomic classification of new betanodavirus isolates by phylogenetic analysis of the coat protein gene suggests a low host-fish species specificity. J. Gen. Virol..

[54] Cutrín J.M., Dopazo C.P., Thiéry R., Leao P., Olveira J.G., Barja J.L., Bandín I. (2007). Emergence of pathogenic betanodaviruses belonging to the SJNNV genogroup in farmed fish species from the Iberian Peninsula. J. Fish Dis..

[55] Olveira J.G., Souto S., Dopazo C.P., Thiéry R., Barja J.L., Bandín I. (2009). Comparative analysis of both genomic segments of betanodaviruses isolated from epizootic outbreaks in farmed fish species provides evidence for genetic reassortment. J. Gen. Virol..

[56] Panzarin V., Fusaro A., Monne I., Cappellozza E., Patarnello P., Bovo G., Capua I., Holmes E.C., Cattoli G. (2012). Molecular epidemiology and evolutionary dynamics of betanodavirus in southern Europe. Infect. Genet. Evol..

[57] Toffan A., Pascoli F., Pretto T., Panzarin V., Abbadi M., Buratin A., Quartesan R., Gijon D., Padros F. (2017). Viral nervous necrosis in gilthead sea bream (*Sparus aurata*) caused by reassortant betanodavirus RGNNV/SJNNV: An emerging threat for Mediterranean aquaculture. Sci. Rep..

[58] Bitchava K., Chassalevris T., Lampou E., Athanassopoulou F., Economou V., Dovas C.I. (2019). Occurrence and molecular characterization of betanodaviruses in fish and invertebrates of the Greek territorial waters. J. Fish Dis..

[59] Mori K., Mangyoku T., Iwamoto T., Arimoto M., Tanaka S., Nakai T. (2003). Serological relationships among genotypic variants of betanodavirus. Dis. Aquat. Organ..

[60] Panzarin V., Toffan A., Abbadi M., Buratin A., Mancin M., Braaen S., Olsen C.M., Bargelloni L., Rimstad E., Cattoli G. (2016). Molecular basis for antigenic diversity of genus Betanodavirus. PLoS ONE.

[61] Barker D.E., MacKinnon A.M., Boston L., Burt M.D.B., Cone D.K., Speare D.J., Griffiths S., Cook M., Ritchie R., Olivier G. (2002). First report of piscine nodavirus infecting wild winter flounder *Pleuronectes americanus* in Passamaquoddy Bay, New Brunswick, Canada. Dis. Aquat. Organ..

[62] Ciulli S., Galletti E., Grodzki M., Alessi A., Battilani M., Prosperi S. (2007). Isolation and genetic characterization of Betanodavirus from wild marine fish from the Adriatic Sea. Vet. Res. Commun..

[63] Gomez D.K., Mori K., Okinaka Y., Nakai T., Park S.C. (2010). Trash fish can be a source of betanodaviruses for cultured marine fish. Aquaculture.

[64] Haddad-Boubaker S., Boughdir W., Sghaier S., Souissi J.B., Megdich A., Dhaouadi R., Amara A., Panzarin V., Fakhfakh E. (2014). Outbreak of viral nervous necrosis in endangered fish species *Epinephelus costae* and *E. Marginatus* in northern Tunisian coasts. Fish Pathol..

[65] Haddad-Boubaker S., Bigarré L., Bouzgarou N., Megdich A., Baud M., Cabon J., Chéhida N.B. (2013). Molecular epidemiology of betanodaviruses isolated from sea bass and sea bream cultured along the Tunisian coasts. Virus Genes.

[66] Moreno P., Olveira J.G., Labella A., Cutrín J.M., Baro J.C., Borrego J.J., Dopazo C.P. (2014). Surveillance of viruses in wild fish populations in areas around the Gulf of Cadiz (South Atlantic Iberian Peninsula). Appl. Environ. Microbiol..

[67] Cherif N., Fatma A. (2017). Nodaviruses in wild fish population collected around aquaculture cage sites from coastal areas of tunisia. Fish. Aquac. J..

[68] Gomez D.K., Okinaka Y., Nakai T., Sato J., Mushiake K., Isshiki T. (2004). PCR-based detection of betanodaviruses from cultured and wild marine fish with no clinical signs. J. Fish Dis..

[69] Sakamoto T., Okinaka Y., Mori K.-I., Sugaya T., Nishioka T., Oka M., Yamashita H., Nakai T. (2008). Phylogenetic analysis of betanodavirus RNA2 identified from wild marine fish in oceanic regions. Fish Pathol..

[70] Ma H., Wen W., Su Y., Feng J., Xu L., Peng C., Guo Z. (2015). Epidemiological characterization of VNNV in hatchery-reared and wild marine fish on Hainan Island, China, and experimental infection of golden pompano (*Trachinotus ovatus*) juveniles. Arch. Virol..

[71] Liu X.D.L., Huang J.N., Weng S.P., Hu X.Q., Chen W.J., Qin Z.D., Dong X.X., Liu X.D.L., Zhou Y., Asim M. (2015). Infections of nervous necrosis virus in wild and cage-reared marine fish from South China Sea with unexpected wide host ranges. J. Fish Dis..

[72] Nishioka T., Sugaya T., Kawato Y., Mori K., Nakai T. (2016). Pathogenicity of striped jack nervous necrosis virus (SJNNV) Isolated from asymptomatic wild Japanese jack mackerel *Trachurus japonicus*. Fish Pathol..

[73] Curtis P.A., Drawbridge M., Iwamoto T., Nakai T., Hedrick R.P., Gendron A.P. (2001). Nodavirus infection of juvenile white seabass, *Atractoscion nobilis*, cultured in southern California: First record of viral nervous necrosis (VNN) in North America. J. Fish Dis..

[74] Munday B.L., Kwang J., Moody N. (2002). Review article Betanodavirus infections of teleost fish: A review. J. Fish Dis..

[75] Yoshikoshi K., Inoue K. (1990). Viral nervous necrosis in hatchery-reared larvae and juveniles of Japanese parrotfish, *Oplegnathus fasciatus* (Temminck & Schlegel). J. Fish Dis..

[76] Muroga K. (1995). Viral and bacterial diseases in larval and juvenile marine fish and shellfish: A review. Fish Pathol..

[77] Skliris G.P., Krondiris J.V., Sideris D.C., Shinn A.P., Starkey W.G., Richards R.H. (2001). Phylogenetic and antigenic characterization of new fish nodavirus isolates from Europe and Asia. Virus Res..

[78] Johnson S.C., Sperker S.A., Leggiadro C.T., Groman D.B., Griffiths S.G., Ritchie R.J., Cook M.D., Cusack R.R. (2002). Identification and characterization of a piscine neuropathy and nodavirus from juvenile Atlantic cod from the Atlantic coast of North America. J. Aquat. Anim. Health.

[79] Mao M.-G., Wen S.-H., Perálvarez-Marín A., Li H., Jiang J.-L., Jiang Z.-Q., Li X., Sun H., Lü H.-Q. (2015). Evidence for and characterization of nervous necrosis virus infection in Pacific cod (*Gadus macrocephalus*). Arch. Virol..

[80] Sethi S.N., Vinod K., Rudhramurthy N., Kokane M.R., Pattnaik P. (2018). Detection of betanodavirus in wild caught fry milk fish, *Chanos chanos*, (Lacepeds 1803). Indian J. Geo Mar. Sci..

[81] Ucko M., Colorni A., Diamant A. (2004). Nodavirus infections in Israeli mariculture. J. Fish Dis..

[82] Zorriehzahra M.E.J., Ghasemi M., Ghiasi M., Karsidani S.H., Bovo G., Nazari A., Adel M., Arizza V., Dhama K. (2016). Isolation and confirmation of viral nervous necrosis (VNN) disease in golden grey mullet (*Liza aurata*) and leaping mullet (*Liza saliens*) in the Iranian waters of the Caspian Sea. Vet. Microbiol..

[83] Sharma S.R.K., Pradeep M.A., Dube P.N., Kumar T.V.A., Kumar R., Swaminathan T.R. (2019). Betanodavirus-associated mortality in Asian seabass (*Lates calcarifer*, Bloch) cultured in indoor tanks and sea cages. Aquac. Int..

[84] Chi S.C., Shieh J.R., Lin S.J. (2003). Genetic and antigenic analysis of betanodaviruses isolated from aquatic organisms in Taiwan. Dis. Aquat. Organ..

[85] Chi S., Lee K., Hwang S. (2001). Investigation of Host Range of Fish Nodavirus in Taiwan. Proceedings of the Tenth International Conference on Diseases of Fish and Shellfish.

[86] Ransangan J., Manin B.O. (2010). Mass mortality of hatchery-produced larvae of Asian seabass, *Lates calcarifer* (Bloch), associated with viral nervous necrosis in Sabah, Malaysia. Vet. Microbiol..

[87] Jung S.J., Miyazaki T., Miyata M., Oishi T. (1996). Histopathological studies on viral nervous necrosis in a new host, Japanese sea bass *Lateolabrax japonicus*. Bullettin Fac. Bioresour..

[88] David R., Tréguier C., Montagnani C., Belliard C., Levy P., Nédélec G., Joufoques V., Remoissenet G., Gueguen Y., Cochennec-Laureau N. (2010). Molecular detection of betanodavirus from the farmed fish, *Platax orbicularis* (Forsskal) (Ephippidae), in French Polynesia. J. Fish Dis..

[89] Ransangan J., Manin B.O., Abdullah A., Roli Z., Sharudin E.F. (2011). Betanodavirus infection in golden pompano, *Trachinotus blochii*, fingerlings cultured in deep-sea cage culture facility in Langkawi, Malaysia. Aquaculture.

[90] Li P., Yu Q., Li F., Qin X., Dong D., Chen B., Qin Q. (2018). First identification of the nervous necrosis virus isolated from cultured golden pompano (*Trachinotus ovatus*) in Guangxi, China. J. Fish Dis..

[91] Abdullah A., Ramli R., Ridzuan M.S.M., Murni M., Hashim S., Sudirwan F., Abdullah S.Z., Mansor N.N., Amira S., Saad M.Z. (2017). The presence of Vibrionaceae, Betanodavirus and Iridovirus in marine cage-cultured fish: Role of fish size, water physicochemical parameters and relationships among the pathogens. Aquac. Rep..

[92] Toffolo V., Negrisolo E., Maltese C., Bovo G., Belvedere P., Colombo L., Valle L.D. (2007). Phylogeny of betanodaviruses and molecular evolution of their RNA polymerase and coat proteins. Mol. Phylogenet. Evol..

[93] Bovo G., Nishizawa T., Maltese C., Borghesan F., Mutinelli F., Montesi F., De Mas S. (1999). Viral encephalopathy and retinopathy of farmed marine fish species in Italy. Virus Res..

[94] Le Breton A., Grisez L., Sweetman J., Ollevier F. (1997). Viral nervous necrosis (VNN) associated with mass mortalities in cage-reared sea bass, *Dicentrarchus labrax* (L.). J. Fish Dis..

[95] Athanassopoulou F., Billinis C., Psychas V., Karipoglou K. (2003). Viral encephalopathy and retinopathy of *Dicentrarchus labrax* (L.) farmed in fresh water in Greece. J. Fish Dis..

[96] Vendramin N., Patarnello P., Toffan A., Panzarin V., Cappellozza E., Tedesco P., Terlizzi A., Terregino C., Cattoli G. (2013). Viral Encephalopathy and Retinopathy in groupers (*Epinephelus* spp.) in southern Italy: A threat for wild endangered species?. BMC Vet. Res..

[97] Binesh C.P., Renuka K., Malaichami N., Greeshma C. (2013). First report of viral nervous necrosis-induced mass mortality in hatchery-reared larvae of clownfish, *Amphiprion sebae* Bleeker. J. Fish Dis..

[98] Oh M.J., Jung S.J., Kim S.R., Rajendran K.V., Kim Y.J., Choi T.J., Kim H.R., Kim J. (2002). Do A fish nodavirus associated with mass mortality in hatchery-reared red drum, *Sciaenops ocellatus*. Aquaculture.

[99] Comps M., Trindade M., Delsert C. (1996). Investigation of fish encephalitis viruses (FEV) expression in marine fishes using DIG-labelled probes. Aquaculture.

[100] Pavoletti E., Prearo M., Ghittino M., Ghittino C. (1998). Casi di encefaloretinopatia in ombrina (*Umbrina cirrosa*) con descrizione della sintomatologia clinica e del quadro anatomoistopatologico. Boll. Soc. Patol. Ittica.

[101] Sugaya T., Mori K., Nishioka T., Masuma S., Oka M., Mushiake K., Okinaka Y., Nakai T. (2009). Genetic heterogeneity of betanodaviruses in juvenile production trials of Pacific bluefin tuna, *Thunnus orientalis* (Temminck & Schlegel). J. Fish Dis..

[102] Chi S.C., Lo C.F., Kou G.H., Chang P.S., Peng S.E., Chen S.N. (1997). Mass mortalities associated with viral nervous necrosis (VNN) disease in two species of hatchery-reared grouper, *Epinephelus fuscogutatus* and *Epinephelus akaara* (Temminck & Schlegel). J. Fish Dis..

[103] Mori K., Nakai T., Nagahara M., Muroga K., Mekuchi T., Kanno T. (1991). A viral disease in hatchery-reared larvae and juveniles of redspotted grouper. Fish Pathol..

[104] Lai Y.-S.S., Murali S., Chiu H.-C.C., Ju H.-Y.Y., Lin Y.-S.S., Chen S.-C.C., Guo I.-C.C., Fang K., Chang C.-Y.Y. (2001). Propagation of yellow grouper nervous necrosis virus (YGNNV) in a new nodavirus-susceptible cell line from yellow grouper, *Epinephelus awoara* (Temminck & Schlegel), brain tissue. J. Fish Dis..

[105] Maeno Y., de la Pena L.D., Cruz-Lacierda E.R. (2002). Nodavirus infection in hatchery-reared orange-spotted grouper *Epinephelus coioides*: First record of viral nervous necrosis in the Philippines. Fish Pathol..

[106] Kara H.M., Chaoui L., Derbal F., Zaidi R., de Boisséson C., Baud M., Bigarré L. (2014). Betanodavirus-associated mortalities of adult wild groupers *Epinephelus marginatus* (Lowe) and *Epinephelus costae* (Steindachner) in Algeria. J. Fish Dis..

[107] Lin C.S., Lu M.W., Tang L., Liu W., Chao C.B., Lin C.J., Krishna N.K., Johnson J.E., Schneemann A. (2001). Characterization of virus-like particles assembled in a recombinant baculovirus system expressing the capsid protein of a fish nodavirus. Virology.

[108] Agnihotri K., Pease B., Chong R. (2016). Molecular analysis of RNA1 and RNA2 sequences from a betanodavirus isolated from giant grouper (*Epinephelus lanceolatus*) in Australia. Virol. Rep..

[109] Valencia J., Grau A., Pretto T., Pons J., Jurado-Rivera J., Castro J., Toffan A., Catanese G. (2019). Viral encephalopathy and retinopathy (VER) disease in *Epinephelus marginatus* from the Balearic Islands marine protected areas. Dis. Aquat. Organ..

[110] Nakai T., Dung N.H., Nishizawa T., Muroga K., Arimoto M., Ootsuki K. (1994). Occurrence of viral nervous necrosis in kelp grouper and tiger puffer. Fish Pathol..

[111] Fukuda Y., Nguyen H.D., Furuhashi M., Nakai T. (1996). Mass Mortality of Cultured Sevenband Grouper, *Epinephelus septemfasciatus*, Associated with Viral Nervous Necrosis. Fish Pathol..

[112] Sohn S.-G., Park M.-A., Oh M.-J., Chun S.-K. (1998). A Fish Nodavirus Isolated from Cultured Sevenband Groupe, *Epinephelus septemfasciatus*. J. Fish Pathol..

[113] Chua F., Loo J., Wee J. (1995). Mass mortality in juvenile greasy grouper, *Epinephelus tauvina*, associated with vacuolating encephalopathy and retinopathy. Dis Asian Aquacult.

[114] Hegde A., Chen C.L., Qin Q.W., Lam T.J., Sin Y.M. (2002). Characterization, pathogenicity and neutralization studies of a nervous necrosis virus isolated from grouper, *Epinephelus tauvina*, in Singapore. Aquaculture.

[115] Khumaidi A., Fadjar M., Iranawati F., Kilawati Y., Yanuhar U. (2019). Mass Mortality Associated with Viral Nervous Necrosis of Hybrid Grouper (*Epinephelus* sp.) Cultured in City of Grouper. Proceedings of the International Conference on Biology and Applied Science (ICOBAS).

[116] Knibb W., Luu G., Premachandra H.K.A., Lu M.-W., Nguyen N.H. (2017). Regional genetic diversity for NNV grouper viruses across the Indo-Asian region—Implications for selecting virus resistance in farmed groupers. Sci. Rep..

[117] Ransangan J., Manin B.O. (2012). Genome analysis of Betanodavirus from cultured marine fish species in Malaysia. Vet. Microbiol..

[118] Zafran, Koesharyani I., Johnny F., Yuasa K., Harada T., Hatai K. (2000). Viral nervous necrosis in humpback grouper *Cromileptes altivelis* larvae and juveniles in Indonesia. Fish Pathol..

[119] Pirarat N., Ponpornpisit A., Traithong T., Nakai T., Katagiri T., Maita M., Endo M. (2009). Nodavirus associated with pathological changes in adult spotted coralgroupers (*Plectropomus maculatus*) in Thailand with viral nervous necrosis. Res. Vet. Sci..

[120] NaveenKumar S., Hassan M.A., Mahmoud M.A., Al-Ansari A., Al-Shwared W.K. (2017). Betanodavirus infection in reared marine fishes along the Arabian Gulf. Aquac. Int..

[121] Kim S.R., Jung S.J., Kim Y.J., Kim J.D., Jung T.S., Choi T.J., Yoshimizu M., Oh M.J. (2002). Phylogenic Comparison of Viral Nervous Necrosis (VNN) Viruses Occurring Seed Production Period. Korean J. Fish. Aquat. Sci..

[122] Grotmol S., Totland G.K., Kvellestad A., Fjell K., Olsen A.B. (1995). Mass mortality of larval and juvenile hatchery-reared halibut (*Hippoglossus hippoglossus* L.) associated with the presence of virus-like particles in vacuolated lesions in the central nervous system and retina. Bull. Eur. Assoc. Fish Pathol..

[123] Grotmol S., Nerland A.H., Biering E., Totland G.K., Nishizawa T. (2000). Characterisation of the capsid protein gene from a nodavirus strain affecting the Atlantic halibut *Hippoglossus hippoglossus* and design of an optimal reverse-transcriptase polymerase chain reaction (RT-PCR) detection assay. Dis. Aquat. Organ..

[124] Starkey W.G., Ireland J.H., Muir K.F., Shinn A., Richards R.H., Ferguson H.W. (2000). Isolation of nodavirus from Scottish farmed halibut, *Hippoglossus hippoglossus* (L). J. Fish Dis..

[125] Pirarat N., Katagiri T., Maita M., Nakai T., Endo M. (2009). Viral encephalopathy and retinopathy in hatchery-reared juvenile thread-sail filefish (*Stephanolepis cirrhifer*). Aquaculture.

[126] Gomez D.K., Lim D.J., Baeck G.W., Youn H.J., Shin N.S., Youn H.Y., Hwang C.Y., Park J.H., Park S.C. (2006). Detection of betanodaviruses in apparently healthy aquarium fishes and invertebrates. J. Vet. Sci..

[127] Kim Y.C., Kwon W.J., Min J.G., Jeong H.D. (2018). Isolation and initial characterization of new betanodaviruses in shellfish. Transbound. Emerg. Dis..

[128] Panzarin V., Patarnello P., Mori A., Rampazzo E., Cappellozza E., Bovo G., Cattoli G. (2010). Development and validation of a real-time TaqMan PCR assay for the detection of betanodavirus in clinical specimens. Arch. Virol..

[129] Castric J., Thiéry R., Jeffroy J., De Kinkelin P., Raymond J.C. (2001). Sea bream *Sparus aurata*, an asymptomatic contagious fish host for nodavirus. Dis. Aquat. Organ..

[130] Berzak R., Scheinin A., Davidovich N., Regev Y., Diga R., Tchernov D., Morick D. (2019). Prevalence of nervous necrosis virus (NNV) and *Streptococcus* species in wild marine fish and crustaceans from the Levantine Basin, Mediterranean Sea. Dis. Aquat. Organ..

[131] Kim Y.C., Kwon W.J., Kim M.S., Kim K.I., Min J.G., Jeong H.D. (2018). High prevalence of betanodavirus barfin flounder nervous necrosis virus as well as red-spotted grouper nervous necrosis virus genotype in shellfish. J. Fish Dis..

[132] Olveira J.G., Souto S., Dopazo C.P., Bandín I. (2013). Isolation of betanodavirus from farmed turbot *Psetta maxima* showing no signs of viral encephalopathy and retinopathy. Aquaculture.

[133] Athanassopoulou F., Billinis C., Prapas T. (2004). Important disease conditions of newly cultured species in intensive freshwater farms in Greece: First incidence of nodavirus infection in *Acipenser* sp. Dis. Aquat. Organ..

[134] Jithendran K.P., Shekhar M.S., Kannappan S., Azad I.S. (2011). Nodavirus infection in freshwater ornamental fishes in India-diagnostic histopathology and nested RT-PCR. Asian Fish. Sci..

[135] Vendramin N., Padrós F., Pretto T., Cappellozza E., Panzarin V., Bovo G., Toffan A., Terregino C. (2012). Viral encephalopathy and retinopathy outbreak in restocking facilities of the endangered freshwater species, *Salaria fluviatilis* (Asso). J. Fish Dis..

[136] Bovo G., Gustinelli A., Quaglio F., Gobbo F., Panzarin V., Fusaro A., Mutinelli F., Caffara M., Fioravanti M. (2011). Viral encephalopathy and retinopathy outbreak in freshwater fish farmed in Italy. Dis. Aquat. Organ..

[137] Bigarré L., Cabon J., Baud M., Heimann M., Body A., Lieffrig F., Castric J. (2009). Outbreak of betanodavirus infection in tilapia, *Oreochromis niloticus* (L.), in fresh water. J. Fish Dis..

[138] Bandín I., Souto S., Cutrín J.M., López-Vázquez C., Olveira J.G., Esteve C., Alcaide E., Dopazo C.P. (2014). Presence of viruses in wild eels *Anguilla anguilla* L, from the Albufera Lake (Spain). J. Fish Dis..

[139] Giacopello C., Foti M., Bottari T., Fisichella V., Barbera G. (2013). Detection of viral encephalopathy and retinopathy virus (VERV) in wild marine fish species of the South Tyrrhenian Sea (Central Mediterranean). J. Fish Dis..

[140] Lopez-Jimena B., Cherif N., Garcia-Rosado E., Infante C., Cano I., Castro D., Hammami S., Borrego J.J., Alonso M.C. (2010). A combined RT-PCR and dot-blot hybridization method reveals the coexistence of SJNNV and RGNNV betanodavirus genotypes in wild meagre (*Argyrosomus regius*). J. Appl. Microbiol..

[141] Dalla Valle L., Zanella L., Patarnello P., Paolucci L., Belvedere P., Colombo L. (2000). Development of a sensitive diagnostic assay for fish nervous necrosis virus based on RT-PCR plus nested PCR. J. Fish Dis..

[142] Nakai T., Mori K., Sugaya T., Nishioka T., Mushiake K., Yamashita H. (2010). Current knowledge on viral nervous necrosis (VNN) and its causative betanodaviruses. Isr. J. Aquac..

[143] García-Rosado E., Cano I., Martín-Antonio B., Labella A., Manchado M., Alonso M.C., Castro D., Borrego J.J. (2007). Co-occurrence of viral and bacterial pathogens in disease outbreaks affecting newly cultured sparid fish. Int. Microbiol..

[144] Maltese C., Bovo G. (2007). MONOGRAFIE Viral encephalopathy and retinopathy Encefalopatia e retinopatia virale. Ittiopatologia.

[145] Toffan A., Panzarin V., Toson M., Cecchettin K., Pascoli F. (2016). Water temperature affects pathogenicity of different betanodavirus genotypes in experimentally challenged *Dicentrarchus labrax*. Dis. Aquat. Organ..

[146] Grotmol S., Bergh Ø., Totland G.K. (1999). Transmission of viral encephalopathy and retinopathy (VER) to yolk-sac larvae of the Atlantic halibut *Hippoglossus hippoglossus*: Occurrence of nodavirus in various organs and a possible route of infection. Dis. Aquat. Organ..

[147] Vendramin N., Toffan A., Mancin M., Cappellozza E., Panzarin V., Bovo G., Cattoli G., Capua I., Terregino C. (2014). Comparative pathogenicity study of ten different betanodavirus strains in experimentally infected European sea bass, *Dicentrarchus labrax* (L.). J. Fish Dis..

[148] Souto S., Olveira J.G., Bandín I. (2015). Influence of temperature on Betanodavirus infection in Senegalese sole (*Solea senegalensis*). Vet. Microbiol..

[149] Tanaka S., Aoki H., Nakai T. (1998). Pathogenicity of the nodavirus detected from diseased sevenband grouper *Epinephelus septemfasciatus*. Fish Pathol..

[150] Totland G.K., Grotmol S., Morita Y., Nishioka T., Nakai T. (1999). Pathogenicity of nodavirus strains from striped jack *Pseudocaranx dentex* and Atlantic halibut *Hippoglossus hippoglossus*, studied by waterborne challenge of yolk-sac larvae of both teleost species. Dis. Aquat. Organ..

[151] Hata N., Okinaka Y., Iwamoto T., Kawato Y., Mori K.I., Nakai T. (2010). Identification of RNA regions that determine temperature sensitivities in betanodaviruses. Arch. Virol..

[152] de Silva S.S., Soto D., Cochrane K., de Young C., Soto D., Bahri T. (2009). Climate change and aquaculture: Potential impacts, adaptation and migraton. Climate Change Implications for Fisheries and Aquaculture: Overview of Current Scientific Knowledge.

[153] Slenning B.D. (2010). Global Climate Change and Implications for Disease Emergence. Vet. Pathol..

[154] Bett B., Kiunga P., Gachohi J., Sindato C., Mbotha D., Robinson T., Lindahl J., Grace D. (2017). Effects of climate change on the occurrence and distribution of livestock diseases. Prev. Vet. Med..

[155] Brand S.P.C., Keeling M.J. (2017). The impact of temperature changes on vector-borne disease transmission: Culicoides midges and bluetongue virus. J. R. Soc. Interface.

[156] Niedbalski W., Fitzner A. (2018). Impact of climate change on the occurrence and distribution of bluetongue in Europe. Med. Weter..

[157] Nguyen H.D., Nakai T., Muroga K. (1996). Progression of striped jack nervous necrosis virus (SJNNV) infection in naturally and experimentally infected striped jack *Pseudocaranx dentex* larvae. Dis. Aquat. Organ..

[158] Mladineo I. (2003). The immunohistochemical study of nodavirus changes in larval, juvenile and adult sea bass tissue. J. Appl. Ichthyol..

[159] Souto S., Olveira J.G., Alonso M.C., Dopazo C.P., Bandín I. (2018). Betanodavirus infection in bath-challenged Solea senegalensis juveniles: A comparative analysis of RGNNV, SJNNV and reassortant strains. J. Fish Dis..

[160] Péducasse S., Castric J., Thiéry R., Jeffroy J., Le Ven A., Baudin Laurencin F. (1999). Comparative study of viral encephalopathy and retinopathy in juvenile sea bass *Dicentrarchus labrax* infected in different ways. Dis. Aquat. Organ..

[161] Grotmol S., Totland G.K., Thorud K., Hjeltnes B.K. (1997). Vacuolating encephalopathy and retinopathy associated with a nodavirus-like agent: A probable cause of mass mortality of cultured larval and juvenile Atlantic halibut *Hippoglossus hippoglossus*. Dis. Aquat. Org..

[162] Tanaka S., Takagi M., Miyazaki T. (2004). Histopathological studies on viral nervous necrosis of sevenband grouper, *Epinephelus septemfasciatus* Thunberg, at the grow-out stage. J. Fish Dis..

[163] Costa J.Z., Thompson K.D. (2016). Understanding the interaction between Betanodavirus and its host for the development of prophylactic measures for viral encephalopathy and retinopathy. Fish Shellfish Immunol..

[164] Skliris G.P., Richards R.H. (1999). Induction of nodavirus disease in seabass, *Dicentrarchus labrax*, using different infection models. Virus Res..

[165] Gjessing M.C., Kvellestad A., Ottesen K., Falk K. (2009). Nodavirus provokes subclinical encephalitis and retinochoroiditis in adult farmed Atlantic cod, *Gadus morhua* L. J. Fish Dis..

[166] Tanaka S., Kuriyama I., Nakai T., Miyazaki T. (2003). Susceptibility of cultured juveniles of several marine fish to the sevenband grouper nervous necrosis virus. J. Fish Dis..

[167] Korsnes K., Devold M., Nerland A.H., Nylund A. (2005). Viral encephalopathy and retinopathy (VER) in Atlantic salmon *Salmo salar* after intraperitoneal challenge with a nodavirus from Atlantic halibut *Hippoglossus hippoglossus*. Dis. Aquat. Organ..

[168] Bitchava K., Xylouri E., Fragkiadaki E., Athanassopoulou F., Papanastassopoulou M., Sabatakou O. (2007). First incidence of clinical signs of nodavirus infection in sea bream, *Sparus auratus* L. Isr. J. Aquac. Bamidgeh.

[169] Huang R., Zhu G., Zhang J., Lai Y., Xu Y., He J., Xie J. (2017). Betanodavirus-like particles enter host cells via clathrin-mediated endocytosis in a cholesterol-, pH- and cytoskeleton-dependent manner. Vet. Res..

[170] Liu W., Hsu C.-H., Hong Y.-R., Wu S.-C., Wang C.-H., Wu Y.-M., Chao C.-B., Lin C.-S. (2005). Early endocytosis pathways in SSN-1 cells infected by dragon grouper nervous necrosis virus. J. Gen. Virol..

[171] Iwamoto T., Nakai T., Mori K., Arimoto M., Furusawa I., Nakai T. (2000). High permissivity of the fish cell line SSN-1 for piscine nodaviruses. Dis. Aquat. Organ..

[172] Chang J.-S., Chi S.-C. (2015). GHSC70 is involved in the cellular entry of nervous necrosis virus. J. Virol..

[173] Krishnan R., Kim J.-O., Kim J.-O., Qadiri S.S.N., Kim S.-J., Oh M.-J. (2019). Immunoglobulin-like cell adhesion molecules, nectins—Characterization, functional prediction and expression profiling from seven-band grouper, *Hyporthodus septemfasciatus*. Aquaculture.

[174] Krishnan R., Qadiri S.S.N., Oh M.-J. (2019). Functional characterization of seven-band grouper immunoglobulin like cell adhesion molecule, Nectin4 as a cellular receptor for nervous necrosis virus. Fish Shellfish Immunol..

[175] Iwamoto T., Okinaka Y., Mise K., Mori K.-I., Arimoto M., Okuno T., Nakai T. (2004). Identification of host-specificity determinants in betanodaviruses by using reassortants between striped jack nervous necrosis virus and sevenband grouper nervous necrosis virus. J. Virol..

[176] Ito Y., Okinaka Y., Mori K.I., Sugaya T., Nishioka T., Oka M., Nakai T. (2008). Variable region of betanodavirus RNA2 is sufficient to determine host specificity. Dis. Aquat. Organ..

[177] Souto S., Olveira J.G., Vázquez-Salgado L., Dopazo C.P., Bandín I. (2018). Betanodavirus infection in primary neuron cultures from sole. Vet. Res..

[178] Ikenaga T., Tatecho Y., Nakai T., Uematsu K. (2002). Betanodavirus as a novel transneuronal tracer for fish. Neurosci. Lett..

[179] Olveira J.G., Soares F., Engrola S., Dopazo C.P., Bandín I. (2008). Antemortem versus postmortem methods for detection of betanodavirus in Senegalese sole (*Solea senegalensis*). J. Vet. Diagn. Investig..

[180] Korsnes K., Karlsbakk E., Devold M., Nerland A.H., Nylund A. (2009). Tissue tropism of nervous necrosis virus (NNV) in Atlantic cod, *Gadus morhua* L., after intraperitoneal challenge with a virus isolate from diseased Atlantic halibut, *Hippoglossus hippoglossus* (L.). J. Fish Dis..

[181] Kim J.-O., Kim S.-J., Kim J.-O., Kim W.-S., Oh M.-J. (2018). Distribution of nervous necrosis virus (NNV) in infected sevenband grouper, *Hyporthodus septemfasciatus* by intramuscular injection or immersion challenge. Aquaculture.

[182] Nguyen H.D., Mushiake K., Nakai T., Muroga K. (1997). Tissue distribution of striped jack nervous necrosis virus (SJNNV) in adult striped jack. Dis. Aquat. Organ..

[183] Azad I.S., Jithendran K.P., Shekhar M.S., Thirunavukkarasu A.R., De La Peña L.D. (2006). Immunolocalisation of nervous necrosis virus indicates vertical transmission in hatchery produced Asian sea bass (*Lates calcarifer* Bloch)—A case study. Aquaculture.

[184] Grove S., Johansen R., Reitan L.J., Press C.M., Dannevig B.H. (2006). Quantitative investigation of antigen and immune response in nervous and lymphoid tissues of Atlantic halibut (*Hippoglossus hippoglossus*) challenged with nodavirus. Fish Shellfish Immunol..

[185] Lopez-Jimena B., Alonso M.C., Thompson K.D., Adams A., Infante C., Castro D., Borrego J.J., Garcia-Rosado E. (2011). Tissue distribution of Red Spotted Grouper Nervous Necrosis Virus (RGNNV) genome in experimentally infected juvenile European seabass (*Dicentrarchus labrax*). Vet. Microbiol..

[186] Mazelet L., Dietrich J., Rolland J.L. (2011). New RT-qPCR assay for viral nervous necrosis virus detection in sea bass, *Dicentrarchus labrax* (L.): Application and limits for hatcheries sanitary control. Fish Shellfish Immunol..

[187] Valero Y., Arizcun M., Esteban M.Á., Bandín I., Olveira J.G., Patel S., Cuesta A., Chaves-Pozo E. (2015). Nodavirus colonizes and replicates in the testis of gilthead seabream and european sea bass modulating its immune and reproductive functions. PLoS ONE.

[188] Su Y., Xu H., Ma H., Feng J., Wen W., Guo Z. (2015). Dynamic distribution and tissue tropism of nervous necrosis virus in juvenile pompano (*Trachinotus ovatus*) during early stages of infection. Aquaculture.

[189] Parameswaran V., Kumar S.R., Ahmed V.P.I., Hameed A.S.S. (2008). A fish nodavirus associated with mass mortality in hatchery-reared Asian Sea bass, *Lates calcarifer*. Aquaculture.

[190] Souto S., Lopez-Jimena B., Alonso M.C., García-Rosado E., Bandín I. (2015). Experimental susceptibility of European sea bass and Senegalese sole to different betanodavirus isolates. Vet. Microbiol..

[191] Hick P., Schipp G., Bosmans J., Humphrey J., Whittington R. (2011). Recurrent outbreaks of viral nervous necrosis in intensively cultured barramundi (*Lates calcarifer*) due to horizontal transmission of betanodavirus and recommendations for disease control. Aquaculture.

[192] Pascoli F., Serra M., Toson M., Pretto T., Toffan A. (2016). Betanodavirus ability to infect juvenile European sea bass, *Dicentrarchus labrax*, at different water salinity. J. Fish Dis..

[193] Aranguren R., Tafalla C., Novoa B., Figueras A. (2002). Experimental transmission of encephalopathy and retinopathy induced by nodavirus to sea bream, *Sparus aurata* L., using different infection models. J. Fish Dis..

[194] Manin B.O., Ransangan J. (2011). Experimental evidence of horizontal transmission of Betanodavirus in hatchery-produced Asian seabass, *Lates calcarifer* and brown-marbled grouper, *Epinephelus fuscoguttatus* fingerling. Aquaculture.

[195] Korsnes K., Karlsbakk E., Nylund A., Nerland A.H. (2012). Horizontal transmission of nervous necrosis virus between turbot *Scophthalmus maximus* and Atlantic cod *Gadus morhua* using cohabitation challenge. Dis. Aquat. Organ..

[196] Arimoto M., Mori K., Nakai T., Muroga K., Furusawa I. (1993). Pathogenicity of the causative agent of viral nervous necrosis disease in striped jack, *Pseudocaranx dentex* (Bloch & Schneider). J. Fish Dis..

[197] Frerichs G.N., Tweedie A., Starkey W.G., Richards R.H. (2000). Temperature, pH and electrolyte sensitivity, and heat, UV and disinfectant inactivation of sea bass (*Dicentrarchus labrax*) neuropathy nodavirus. Aquaculture.

[198] Skliris G.P., Richards R.H. (1998). Assessment of the susceptibility of the brine shrimp *Artemia salina* and rotifer *Brachionus plicatilis* to experimental nodavirus infections. Aquaculture.

[199] Vazquez-Salgado L., Olveira J.G., Dopazo C.P., Bandín I. Live food can play a role in nervous necrosis virus (NNV) transmission in marine fish hatcheries (manuscript in preparation).

[200] Gomez D.K., Baeck G.W., Kim J.H., Choresca C.H., Park S.C. (2008). Molecular detection of betanodaviruses from apparently healthy wild marine invertebrates. J. Invertebr. Pathol..

[201] Volpe E., Grodzki M., Panzarin V., Guercio A., Purpari G., Serratore P., Ciulli S. (2018). Detection and molecular characterization of betanodaviruses retrieved from bivalve molluscs. J. Fish Dis..

[202] Fichi G., Cardeti G., Perrucci S., Vanni A., Cersini A., Lenzi C., De Wolf T., Fronte B., Guarducci M., Susini F. (2015). Skin lesion-associated pathogens from *Octopus vulgaris*: First detection of *Photobacterium swingsii*, *Lactococcus garvieae* and betanodavirus. Dis. Aquat. Organ..

[203] Arimoto M., Mushiake K., Mizuta Y., Nakai T., Muroge K., Furusawa I. (1992). Detection of Striped Jack Nervous Necrosis Virus (SJNNV) by Enzyme-Linked Immunosorbent Assay (ELISA). Fish Pathol..

[204] Yoshimizu M., Suzuki K., Nishizawa T., Winton J.R., Ezura Y. Antibody Screening for the Identification of Nervous Necrosis Carriers in Flounder Broodstock. Proceedings of the NRIA International Workshop on New Approaches to Viral Diseases of Aquatic Animals.

[205] Breuil G., Pépin J.F.P., Boscher S., Thiéry R. (2002). Experimental vertical transmission of nodavirus from broodfish to eggs and larvae of the sea bass, *Dicentrarchus labrax* (L.). J. Fish Dis..

[206] Mori K., Mushiake K., Arimoto M. (1998). Control measures for viral nervous necrosis in striped jack. Fish Pathol..

[207] Watanabe K., Nishizawa T., Yoshimizu M. (2000). Selection of brood stock candidates of barfin flounder using an ELISA system with recombinant protein of barfin flounder nervous necrosis virus. Dis. Aquat. Organ..

[208] Jaramillo D., Hick P., Whittington R.J. (2017). Age dependency of nervous necrosis virus infection in barramundi *Lates calcarifer* (Bloch). J. Fish Dis..

[209] Juniar E., Kurniasih K., Sumiarto B. (2018). Risk factors of a viral nervous necrosis disease in grouper (*Epinephelus* spp.) cultured in Bintan district, Indonesia. Vet. World.

[210] Verrier E.R., Langevin C., Benmansour A., Boudinot P. (2011). Early antiviral response and virus-induced genes in fish. Dev. Comp. Immunol..

[211] Zou J., Secombes C.J. (2011). Teleost fish interferons and their role in immunity. Dev. Comp. Immunol..

[212] Chen Y.-M.M., Wang T.-Y.Y., Chen T.-Y.Y. (2014). Immunity to betanodavirus infections of marine fish. Dev. Comp. Immunol..

[213] Scapigliati G., Buonocore F., Randelli E., Casani D., Meloni S., Zarletti G., Tiberi M., Pietretti D., Boschi I., Manchado M. (2010). Cellular and molecular immune responses of the sea bass (*Dicentrarchus labrax*) experimentally infected with betanodavirus. Fish Shellfish Immunol..

[214] Øvergård A.C., Nerland A.H., Fiksdal I.U., Patel S. (2012). Atlantic halibut experimentally infected with nodavirus shows increased levels of T-cell marker and IFNγ transcripts. Dev. Comp. Immunol..

[215] Chen C.W., Wu M.S., Huang Y.J., Cheng C.A., Chang C.Y. (2015). Recognition of linear B-cell epitope of Betanodavirus coat protein by RG-M18 neutralizing mAB inhibits giant grouper nervous necrosis virus (GGNNV) infection. PLoS ONE.

[216] Wu Y.C.C., Chi S.C.C. (2006). Persistence of betanodavirus in Barramundi brain (BB) cell line involves the induction of Interferon response. Fish Shellfish Immunol..

[217] Huang Y., Ouyang Z., Wang W., Yu Y., Li P., Zhou S., Wei S., Wei J., Huang X., Qin Q. (2015). Antiviral role of grouper STING against iridovirus infection. Fish Shellfish Immunol..

[218] Huang R., Zhou Q., Shi Y., Zhang J., He J., Xie J. (2018). Protein A from orange-spotted grouper nervous necrosis virus triggers type I interferon production in fish cell. Fish Shellfish Immunol..

[219] Poisa-Beiro L., Dios S., Montes A., Aranguren R., Figueras A., Novoa B. (2008). Nodavirus increases the expression of Mx and inflammatory cytokines in fish brain. Mol. Immunol..

[220] Moreno P., Lopez-Jimena B., Randelli E., Scapigliati G., Buonocore F., Garcia-Rosado E., Borrego J.J., Alonso M.C. (2018). Immuno-related gene transcription and antibody response in nodavirus (RGNNV and SJNNV)-infected European sea bass (*Dicentrarchus labrax* L.). Fish Shellfish Immunol..

[221] Moreno P., Souto S., Leiva-rebollo R., Borrego J.J., Bandín I., Alonso M.C. (2019). Capsid amino acids at positions 247 and 270 are involved in the virulence of betanodaviruses to European sea bass. Sci. Rep..

[222] Álvarez-Torres D., Podadera A.M., Alonso M.C., Bandín I., Béjar J., García-Rosado E. (2017). Molecular characterization and expression analyses of the *Solea senegalensis* interferon-stimulated gene 15 (isg15) following NNV infections. Fish Shellfish Immunol..

[223] Wu Y., Zhou Y., Cao Z., Sun Y., Chen Y., Xiang Y., Wang L., Zhang S., Guo W. (2019). Comparative analysis of the expression patterns of IL-1β, IL-11, and IL-34 in golden pompano (*Trachinotus ovatus*) following different pathogens challenge. Fish Shellfish Immunol..

[224] Chaves-Pozo E., Guardiola F.A., Meseguer J., Esteban M.A., Cuesta A. (2012). Nodavirus infection induces a great innate cell-mediated cytotoxic activity in resistant, gilthead seabream, and susceptible, European sea bass, teleost fish. Fish Shellfish Immunol..

[225] Kai Y.-H.H., Wu Y.-C.C., Chi S.-C.C. (2014). Immune gene expressions in grouper larvae (*Epinephelus coioides*) induced by bath and oral vaccinations with inactivated betanodavirus. Fish Shellfish Immunol..

[226] Jung J.W., Lee J.S., Kim J., Im S.P., Kim S.W., Lazarte J.M.S., Kim Y.R., Chun J.H., Ha M.W., Kim N.N. (2020). Involvement of CD4-1T cells in the cellular immune response of olive flounder (*Paralichthys olivaceus*) against viral hemorrhagic septicemia virus (VHSV) and nervous necrosis virus (NNV) infection. Dev. Comp. Immunol..

[227] Ozsolak F., Milos P.M. (2011). RNA sequencing: Advances, challenges and opportunities Fatih. Nat. Rev. Genet..

[228] Lu M.-W.W., Ngou F.-H.H., Chao Y.-M.M., Lai Y.-S.S., Chen N.-Y.Y., Lee F.-Y.Y., Chiou P.P. (2012). Transcriptome characterization and gene expression of *Epinephelus* spp in endoplasmic reticulum stress-related pathway during betanodavirus infection in vitro. BMC Genom..

[229] Liu P., Wang L., Kwang J., Yue G.H., Wong S.M. (2016). Transcriptome analysis of genes responding to NNV infection in Asian seabass epithelial cells. Fish Shellfish Immunol..

[230] Chen W., Yi L., Feng S., Liu X., Asim M., Zhou Y., Lan J., Jiang S., Tu J., Lin L. (2017). Transcriptomic profiles of striped snakehead fish cells (SSN-1) infected with red-spotted grouper nervous necrosis virus (RGNNV) with an emphasis on apoptosis pathway. Fish Shellfish Immunol..

[231] Chaves-Pozo E., Valero Y., Esteve-Codina A., Gómez-Garrido J., Dabad M., Alioto T., Meseguer J., Esteban M.Á., Cuesta A. (2017). Innate cell-mediated cytotoxic activity of European sea bass leucocytes against nodavirus-infected cells: A Functional and RNA-seq Study. Sci. Rep..

[232] Chaves-Pozo E., Bandín I., Olveira J.G., Esteve-Codina A., Gómez-Garrido J., Dabad M., Alioto T., Ángeles Esteban M., Cuesta A. (2019). European sea bass brain DLB-1cell line is susceptible to nodavirus: A transcriptomic study. Fish Shellfish Immunol..

[233] Xiang Y., Jia P., Liu W., Yi M., Jia K. (2019). Comparative transcriptome analysis reveals the role of p53 signalling pathway during red-spotted grouper nervous necrosis virus infection in *Lateolabrax japonicus* brain cells. J. Fish Dis..

[234] Krasnov A., Kileng Ø., Skugor S., Jørgensen S.M., Afanasyev S., Timmerhaus G., Sommer A.I., Jensen I. (2013). Genomic analysis of the host response to nervous necrosis virus in Atlantic cod (*Gadus morhua*) brain. Mol. Immunol..

[235] Wang Y.D., Rajanbabu V., Chen J.Y. (2015). Transcriptome analysis of medaka following epinecidin-1 and TH1-5 treatment of NNV infection. Fish Shellfish Immunol..

[236] Tso C.-H.H., Lu M.-W.W. (2018). Transcriptome profiling analysis of grouper during nervous necrosis virus persistent infection. Fish Shellfish Immunol..

[237] Labella A.M., Garcia-Rosado E., Bandín I., Dopazo C.P., Castro D., Alonso M.C., Borrego J.J. (2018). Transcriptomic profiles of Senegalese sole infected with nervous necrosis virus reassortants presenting different degree of virulence. Front. Immunol..

[238] Wang L., Tian Y., Cheng M., Li Z., Li S., Wu Y., Zhang J., Ma W., Li W., Pang Z. (2019). Transcriptome comparative analysis of immune tissues from asymptomatic and diseased *Epinephelus moara* naturally infected with nervous necrosis virus. Fish Shellfish Immunol..

[239] Kim J.O., Kim J.O., Kim W.S., Oh M.J. (2017). Characterization of the transcriptome and gene expression of brain tissue in sevenband grouper (*Hyporthodus septemfasciatus*) in response to NNV infection. Genes.

[240] Barber D.L., Wherry E.J., Masopust D., Zhu B., Allison J.P., Sharpe A.H., Freeman G.J., Ahmed R. (2006). Restoring function in exhausted CD8 T cells during chronic viral infection. Nature.

[241] Okagawa T., Konnai S., Deringer J.R., Ueti M.W., Scoles G.A., Murata S., Ohashi K., Brown W.C. (2016). Cooperation of PD-1 and LAG-3 contributes to T-cell exhaustion in Anaplasma marginale-infected cattle. Infect. Immun..

[242] Chiang Y.-H., Wu Y.-C., Chi S.-C. (2017). Interleukin-1β secreted from betanodavirus-infected microglia caused the death of neurons in giant grouper brains. Dev. Comp. Immunol..

[243] Snieszko S.F. (1973). Recent advances in scientific knowledge and developments pertaining to diseases of fishes. Adv. Vet. Sci. Comp. Med..

[244] Souto S., Mérour E., Biacchesi S., Brémont M., Olveira J.G., Bandín I. (2015). In Vitro and in vivo characterization of molecular determinants of virulence in reassortant betanodavirus. J. Gen. Virol..

[245] Doan Q.K., Vandeputte M., Chatain B., Morin T., Allal F. (2017). Viral encephalopathy and retinopathy in aquaculture: A review. J. Fish Dis..

[246] Gomez D.K., Baeck G.W., Kim J.H., Choresca C.H., Park S.C. (2008). Genetic analysis of betanodaviruses in subclinically infected aquarium fish and invertebrates. Curr. Microbiol..

[247] Ciulli S., Grodzki M., Bignami G., Serratore P., Prosperi S. (2010). Molecular detection and genetic analysis of betanodaviruses in bivalve mollusks. J. Biotechnol..

[248] Fichi G., Cardeti G., Cersini A., Mancusi C., Guarducci M., Di Guardo G., Terracciano G. (2016). Bacterial and viral pathogens detected in sea turtles stranded along the coast of Tuscany, Italy. Vet. Microbiol..

[249] Munday B.L., Langdon J.S., Hyatt A., Humphrey J.D. (1992). Mass mortality associated with a viral-induced vacuolating encephalopathy and retinopathy of larval and juvenile barramundi, *Lates calcarifer* Bloch. Aquaculture.

[250] Chi S.C., Hu W.W., Lo B.J. (1999). Establishment and characterization of a continuous cell line (GF-1) derived from grouper, *Epinephelus coioides* (Hamilton): A cell line susceptible to grouper nervous necrosis virus (GNNV). J. Fish Dis..

[251] Wen C.M., Lee C.W., Wang C.S., Cheng Y.H., Huang H.Y. (2008). Development of two cell lines from *Epinephelus coioides* brain tissue for characterization of betanodavirus and megalocytivirus infectivity and propagation. Aquaculture.

[252] Sarath Babu V., Abdul Majeed S., Nambi K.S.N., Taju G., Madan N., Sundar Raj N., Sahul Hameed A.S. (2013). Comparison of betanodavirus replication efficiency in ten Indian fish cell lines. Arch. Virol..

[253] Ma H., Cheng C., Su Y., Deng Y., Feng J., Guo Z. (2019). Propagations of grouper (*Epinephelus* sp.) viruses in a new fibroblast-like cell line from orange spotted grouper (*E. coioides*) brain. Aquaculture.

[254] Liu X.F., Wu Y.H., Wei S.N., Wang N., Li Y.Z., Zhang N.W., Li P.F., Qin Q.W., Chen S.L. (2018). Establishment and characterization of a brain-cell line from kelp grouper *Epinephelus moara*. J. Fish Biol..

[255] Le Y., Li Y., Jin Y., Jia P., Jia K., Yi M. (2017). Establishment and characterization of a brain cell line from sea perch, *Lateolabrax japonicus*. Vitr. Cell. Dev. Biol. Anim..

[256] Tu J., Chen W., Fu X., Lin Q., Chang O., Zhao L., Lan J., Li N., Lin L. (2016). Susceptibility of Chinese perch brain (CPB) Cell and Mandarin fish to red-spotted grouper nervous necrosis virus (RGNNV) Infection. Int. J. Mol. Sci..

[257] Adachi K., Sumiyoshi K., Ariyasu R., Yamashita K., Zenke K., Okinaka Y. (2010). Susceptibilities of medaka (*Oryzias latipes*) cell lines to a betanodavirus. Virol. J..

[258] Zhou L., Li P., Liu J., Ni S., Yu Y., Yang M., Wei S., Qin Q. (2017). Establishment and characterization of a mid-kidney cell line derived from golden pompano *Trachinotus ovatus*, a new cell model for virus pathogenesis and toxicology studies. Vitr. Cell. Dev. Biol. Anim..

[259] Wang R.R., Zhang N., Wang R.R., Wang S., Wang N. (2017). Two skin cell lines from wild-type and albino Japanese flounder (*Paralichthys olivaceus*): Establishment, characterization, virus susceptibility, efficient transfection, and application to albinism study. Fish Physiol. Biochem..

[260] Li P., Zhou L., Wei S., Yang M., Ni S., Yu Y., Cai J., Qin Q. (2017). Establishment and characterization of a cell line from the head kidney of golden pompano *Trachinotus ovatus* and its application in toxicology and virus susceptibility. J. Fish Biol..

[261] Sahul Hameed A.S., Parameswaran V., Shukla R., Bright Singh I.S., Thirunavukkarasu A.R., Bhonde R.R. (2006). Establishment and characterization of India’s first marine fish cell line (SISK) from the kidney of sea bass (*Lates calcarifer*). Aquaculture.

[262] Parameswaran V., Shukla R., Bhonde R.R., Sahul Hameed A.S. (2006). Splenic cell line from sea bass, *Lates calcarifer*: Establishment and characterization. Aquaculture.

[263] Chi S., Wu Y., Cheng T. (2005). Persistent infection of betanodavirus in a novel cell line derived from the brain tissue of barramundi *Lates calcarifer*. Dis. Aquat. Organ..

[264] Wen C.M. (2016). Characterization and viral susceptibility of a brain cell line from brown-marbled grouper *Epinephelus fuscoguttatus* (Forsskål) with persistent betanodavirus infection. J. Fish Dis..

[265] Adachi K., Ichinose T., Watanabe K., Kitazato K., Kobayashi N. (2008). Potential for the replication of the betanodavirus redspotted grouper nervous necrosis virus in human cell lines. Arch. Virol..

[266] Takizawa N., Adachi K., Ichinose T., Kobayashi N. (2008). Efficient propagation of betanodavirus in a murine astrocytoma cell line. Virus Res..

[267] (2000). Chapter 2.2.2. Viral encephalopathy and retinopathy. OIE Diagnostic Manual for Aquatic Animal Diseases.

[268] Kuo H.C., Wang T.Y., Chen P.P., Chen Y.M., Chuang H.C., Chen T.Y. (2011). Real-time quantitative PCR assay for monitoring of nervous necrosis virus infection in grouper aquaculture. J. Clin. Microbiol..

[269] Kim J.-O., Kim J.-O., Kim W.-S., Oh M.-J. (2016). Development and application of quantitative detection method for nervous necrosis virus (NNV) isolated from sevenband grouper *Hyporthodus septemfasciatus*. Asian Pac. J. Trop. Med..

[270] Nishizawa T., Muroga K., Arimoto M. (1996). Failure of the Polymerase Chain Reaction (PCR) Method to Detect Striped Jack Nervous Necrosis Virus (SJNNV) in Striped Jack *Pseudocaranx dentex* Selected as Spawners. J. Aquat. Anim. Health.

[271] Thiery R., Raymond J.-C., Castric J. (1999). Natural outbreak of viral encephalopathy and retinopathy in juvenile sea bass, *Dicentrarchus labrax*: Study by nested reverse transcriptase–polymerase chain reaction. Virus Res..

[272] Nishizawa T., Mori K., Nakai T., Furusawa I., Muroga K. (1994). Polymerase chain reaction (PCR) amplification of RNA of striped jack nervous necrosis virus (SJNNV). Dis. Aquat. Organ..

[273] Dalla Valle L., Toffolo V., Lamprecht M., Maltese C., Bovo G., Belvedere P., Colombo L. (2005). Development of a sensitive and quantitative diagnostic assay for fish nervous necrosis virus based on two-target real-time PCR. Vet. Microbiol..

[274] Nerland A.H., Skaar C., Eriksen T.B., Bleie H. (2007). Detection of nodavirus in seawater from rearing facilities for Atlantic halibut *Hippoglossus hippoglossus* larvae. Dis. Aquat. Organ..

[275] Hick P., Whittington R.J. (2010). Optimisation and validation of a real-time reverse transcriptase-polymerase chain reaction assay for detection of betanodavirus. J. Virol. Methods.

[276] Baud M., Cabon J., Salomoni A., Toffan A., Panzarin V., Bigarré L. (2015). First generic one step real-time Taqman RT-PCR targeting the RNA1 of betanodaviruses. J. Virol. Methods.

[277] Toubanaki D.K., Margaroni M., Karagouni E. (2015). Development of a novel allele-specific PCR method for rapid assessment of nervous necrosis virus genotypes. Curr. Microbiol..

[278] Toubanaki D.K., Karagouni E. (2017). Genotype-specific real-time PCR combined with high-resolution melting analysis for rapid identification of red-spotted grouper nervous necrosis virus. Arch. Virol..

[279] Thiéry R., Arnauld C., Delsert C. (1999). Two isolates of sea bass, *Dicentrarchus labrax* L., nervous necrosis virus with distinct genomes. J. Fish Dis..

[280] Bigarré L., Baud M., Cabon J., Crenn K., Castric J. (2010). New PCR probes for detection and genotyping of piscine betanodaviruses. J. Fish Dis..

[281] Toubanaki D.K., Margaroni M., Karagouni E. (2015). Nanoparticle-based lateral flow biosensor for visual detection of fish nervous necrosis virus amplification products. Mol. Cell. Probes.

[282] Ferreira I.A., Costa J.Z., Macchia V., Dawn Thompson K., Baptista T. (2019). Detection of Betanodavirus in experimentally infected European seabass (*Dicentrarchus labrax*, Linnaeus 1758) using non-lethal sampling methods. J. Fish Dis..

[283] Starkey G.W., Millar R.M., Jenkins M.E., Ireland J.H., Muir K.F., Richards R.H. (2004). Detection of piscine nodaviruses by real-time nucleic acid sequence based amplification (NASBA). Dis. Aquat. Organ..

[284] Xu H.D., Feng J., Guo Z.X., Ou Y.J., Wang J.Y. (2010). Detection of red-spotted grouper nervous necrosis virus by loop-mediated isothermal amplification. J. Virol. Methods.

[285] Su Z.D., Shi C.Y., Huang J., Shen G.M., Li J., Wang S.Q., Fan C. (2015). Establishment and application of cross-priming isothermal amplification coupled with lateral flow dipstick (CPA-LFD) for rapid and specific detection of red-spotted grouper nervous necrosis virus. Virol. J..

[286] Mekata T., Satoh J., Inada M., Dinesh S., Harsha P., Itami T., Sudhakaran R. (2015). Development of simple, rapid and sensitive detection assay for grouper nervous necrosis virus using real-time loop-mediated isothermal amplification. J. Fish Dis..

[287] Huang B., Tan C., Chang S.F., Munday B., Mathew J.A., Ngoh G.H., Kwang J. (2001). Detection of nodavirus in barramundi, *Lates calcarifer* (Bloch), using recombinant coat protein-based ELISA and RT-PCR. J. Fish Dis..

[288] Shieh J., Chi S. (2005). Production of monoclonal antibodies against grouper nervous necrosis virus (GNNV) and development of an antigen capture ELISA. Dis. Aquat. Organ..

[289] Nuñez-Ortiz N., Pascoli F., Picchietti S., Buonocore F., Bernini C., Toson M., Scapigliati G., Toffan A. (2016). A formalin-inactivated immunogen against viral encephalopathy and retinopathy (VER) disease in European sea bass (*Dicentrarchus labrax*): Immunological and protection effects. Vet. Res..

[290] Gye H.J., Oh M.-J., Nishizawa T. (2018). Lack of nervous necrosis virus (NNV) neutralizing antibodies in convalescent sevenband grouper *Hyporthodus septemfasciatus* after NNV infection. Vaccine.

[291] Lopez-Jimena B., Garcia-Rosado E., Thompson K.D., Adams A., Infante C., Borrego J.J., Alonso M.C. (2012). Distribution of red-spotted grouper nervous necrosis virus (RGNNV) antigens in nervous and non-nervous organs of European seabass (*Dicentrarchus labrax*) during the course of an experimental challenge. J. Vet. Sci..

[292] Tarrab K., Ravid-Peretz S., Ucko M. (2019). Immunoserology of European seabass (*Dicentrarchus labrax*) and white grouper (*Epinephelus aeneus*) as a non-lethal diagnostic tool for viral nervous necrosis. Aquac. Int..

[293] Choi B., Gye H.J.H., Oh M.M.-J., Nishizawa T. (2014). Cell culture medium inhibits antigen binding used in an ELISA for detection of antibodies against nervous necrosis virus. J. Aquat. Anim. Health.

[294] Jaramillo D., Hick P., Deece K., Tweedie A., Kirkland P., Arzey E., Whittington R.J. (2016). Comparison of ELISA formats for detection of antibodies specific for nervous necrosis virus (Betanodavirus) in the serum of immunized barramundi *Lates calcarifer* and Australian bass *Macquaria novemaculeata*. Aquaculture.

[295] Gye H.J., Nishizawa T. (2018). Reducing background optical density in enzyme-linked immunosorbent assay for detecting nervous necrosis virus (NNV)-specific IgM by immobilizing fish sera. Aquaculture.

[296] Zhou L., Li P., Ni S., Yu Y., Yang M., Wei S., Qin Q. (2017). Rapid and sensitive detection of redspotted grouper nervous necrosis virus (RGNNV) infection by aptamer-coat protein-aptamer sandwich enzyme-linked apta-sorbent assay (ELASA). J. Fish Dis..

[297] Kim J.O., Kim J.O., Kim S.J., Kim W.S., Oh M.J. (2018). Development of double labeling in situ hybridization using RNA probes for genome detection of nervous necrosis virus (NNV). Mol. Cell. Probes.

[298] Eivazzadeh-Keihan R., Pashazadeh-Panahi P., Mahmoudi T., Chenab K.K., Baradaran B., Hashemzaei M., Radinekiyan F., Mokhtarzadeh A., Maleki A. (2019). Dengue virus: A review on advances in detection and trends—From conventional methods to novel biosensors. Microchim. Acta.

[299] Lee J.H., Oh B.K., Choi J.W. (2015). Development of a HIV-1 virus detection system based on nanotechnology. Sensors.

[300] Peng X., Luo G., Wu Z., Wen W., Zhang X., Wang S. (2019). Fluorescent-magnetic-catalytic nanospheres for dual-modality detection of H9N2 avian influenza virus. ACS Appl. Mater. Interfaces.

[301] Ochmann S.E., Vietz C., Trofymchuk K., Acuna G.P., Lalkens B., Tinnefeld P. (2017). Optical nanoantenna for single molecule-based detection of Zika Virus nucleic acids without molecular multiplication. Anal. Chem..

[302] Sabzehparvar F., Rahmani Cherati T., Mohsenifar A., Roodbar Shojaei T., Tabatabaei M. (2019). Immobilization of gold nanoparticles with rhodamine to enhance the fluorescence resonance energy transfer between quantum dots and rhodamine; new method for downstream sensing of infectious bursal disease virus. Spectrochim. Acta Part A Mol. Biomol. Spectrosc..

[303] Takemura K., Lee J., Suzuki T., Hara T., Abe F., Park E.Y. (2019). Ultrasensitive detection of norovirus using a magnetofluoroimmunoassay based on synergic properties of gold/magnetic nanoparticle hybrid nanocomposites and quantum dots. Sens. Actuators B Chem..

[304] Wang C., Wang C., Wang X., Wang K., Zhu Y., Rong Z., Wang W., Xiao R., Wang S. (2019). Magnetic SERS strip for sensitive and simultaneous detection of respiratory viruses. ACS Appl. Mater. Interfaces.

[305] Yadavalli T., Shukla D. (2017). Role of metal and metal oxide nanoparticles as diagnostic and therapeutic tools for highly prevalent viral infections. Nanomed. Nanotechnol. Biol. Med..

[306] Zhang T., Tian F., Long L., Liu J., Wu X. (2018). Diagnosis of rubella virus using antigen- conjugated Au @ Pt nanorods as nanozyme probe. Int. J. Nanomed..

[307] Cheng X., Chen G., Rodriguez W.R. (2009). Micro- and nanotechnology for viral detection. Anal. Bioanal. Chem..

[308] Tram D.T.N., Wang H., Sugiarto S., Li T., Ang W.H., Lee C., Pastorin G. (2016). Advances in nanomaterials and their applications in point of care (POC) devices for the diagnosis of infectious diseases. Biotechnol. Adv..

[309] Chavan S.G., Kim D., Hwang J., Choi Y., Hong J.W., Kim J., Lee M.H., Hwang M.P., Choi J. (2019). Enhanced detection of infectious pancreatic necrosis virus via lateral flow chip and fluorometric biosensors based on self-assembled protein nanoprobes. ACS Sens..

[310] Arimoto M., Sato J., Maruyama K., Mimura G., Furusawa I. (1996). Effect of chemical and physical treatments on the inactivation of striped jack nervous necrosis virus (SJNNV). Aquaculture.

[311] Adachi K., Ichinose T., Takizawa N., Watanabe K., Kitazato K., Kobayashi N. (2007). Inhibition of betanodavirus infection by inhibitors of endosomal acidification. Arch. Virol..

[312] Kai Y.H., Su H.M., Tai K.T., Chi S.C. (2010). Vaccination of grouper broodfish (*Epinephelus tukula*) reduces the risk of vertical transmission by nervous necrosis virus. Vaccine.

[313] Wang Y.D., Kung C.W., Chen J.Y. (2010). Antiviral activity by fish antimicrobial peptides of epinecidin-1 and hepcidin 1-5 against nervous necrosis virus in medaka. Peptides.

[314] Huang Y.C., Han Y.S. (2014). Determining anti-betanodavirus compounds through a GF-1 cell-based screening platform. Antiviral Res..

[315] Huang Y.-C., Lin T.-S., Peng C., Chan N.-L., Han Y.-S. (2016). Strong inhibition of betanodavirus replication by ribavirin targeting RNA-dependent RNA polymerase. J. Fish Dis..

[316] Zhou L., Li P., Yang M., Yu Y., Huang Y., Wei J., Wei S., Qin Q. (2016). Generation and characterization of novel DNA aptamers against coat protein of grouper nervous necrosis virus (GNNV) with antiviral activities and delivery potential in grouper cells. Antiviral Res..

[317] Zhou Q., Zhang J., Huang R., Huang S., Wu Y., Huang L., He J., Xie J. (2019). An affinity peptide exerts antiviral activity by strongly binding nervous necrosis virus to block viral entry. Fish Shellfish Immunol..

[318] Morick D., Saragovi A. (2017). Inhibition of nervous necrosis virus by ribavirin in a zebrafish larvae model. Fish Shellfish Immunol..

[319] Sushila N., Hameed A.S.S., Prasad K.P., Majeed S.A., Tripathi G. (2018). In vitro screening of selected antiviral drugs against betanodavirus. J. Virol. Methods.

[320] Sieczkarski S.B., Whittaker G.R. (2002). Influenza virus can enter and infect cells in the absence of clathrin-mediated endocytosis. J. Virol..

[321] Zhu S., Li J., Huang A.-G., Huang J.-Q., Huang Y.-Q., Wang G.-X. (2019). Anti-betanodavirus activity of isoprinosine and improved efficacy using carbon nanotubes based drug delivery system. Aquaculture.

[322] Zhu S., Huang A.G., Luo F., Li J., Li J., Zhu L., Zhao L., Zhu B., Ling F., Wang G.X. (2019). Application of Virus Targeting Nanocarrier Drug Delivery System in Virus-Induced Central Nervous System Disease Treatment. ACS Appl. Mater. Interfaces.

[323] Wang Y.D., Kung C.W., Chi S.C., Chen J.Y. (2010). Inactivation of nervous necrosis virus infecting grouper (*Epinephelus coioides*) by epinecidin-1 and hepcidin 1-5 antimicrobial peptides, and downregulation of Mx2 and Mx3 gene expressions. Fish Shellfish Immunol..

[324] Darmostuk M., Rimpelova S., Gbelcova H., Ruml T. (2015). Current approaches in SELEX: An update to aptamer selection technology. Biotechnol. Adv..

[325] Ramalingam D., Duclair S., Datta S.A.K., Ellington A., Rein A., Prasad V.R. (2011). RNA aptamers directed to human immunodeficiency virus type 1 Gag polyprotein bind to the matrix and nucleocapsid domains and inhibit virus production. J. Virol..

[326] Ditzler M.A., Bose D., Shkriabai N., Marchand B., Sarafianos S.G., Kvaratskhelia M., Burke D.H. (2011). Broad-spectrum aptamer inhibitors of HIV reverse transcriptase closely mimic natural substrates. Nucleic Acids Res..

[327] Yamashita H., Fujita Y., Kawakami H., Nakai T. (2005). The efficacy of inactivated virus vaccine against viral nervous necrosis (VNN). Fish Pathol..

[328] Kai Y.H., Chi S.C. (2008). Efficacies of inactivated vaccines against betanodavirus in grouper larvae (*Epinephelus coioides*) by bath immunization. Vaccine.

[329] Pakingking R., Bautista N.B., de Jesus-Ayson E.G., Reyes O. (2010). Protective immunity against viral nervous necrosis (VNN) in brown-marbled grouper (*Epinephelus fuscogutattus*) following vaccination with inactivated betanodavirus. Fish Shellfish Immunol..

[330] Cheng Y.-K.K., Wu Y.-C.C., Chi S.-C.C. (2017). Humoral and cytokine responses in giant groupers after vaccination and challenge with betanodavirus. Dev. Comp. Immunol..

[331] Pakingking R., de Jesus-Ayson E.G., Reyes O., Brian Bautista N. (2018). Immunization regimen in Asian sea bass (*Lates calcarifer*) broodfish: A practical strategy to control vertical transmission of nervous necrosis virus during seed production. Vaccine.

[332] Valero Y., Mokrani D., Chaves-Pozo E., Arizcun M., Oumouna M., Meseguer J., Esteban M.Á., Cuesta A. (2018). Vaccination with UV-inactivated nodavirus partly protects European sea bass against infection, while inducing few changes in immunity. Dev. Comp. Immunol..

[333] Cho S.Y., Kim H.J., Lan N.T., Han H.-J., Lee D.-C., Hwang J.Y., Kwon M.-G., Kang B.K., Han S.Y., Moon H. (2017). Oral vaccination through voluntary consumption of the convict grouper *Epinephelus septemfasciatus* with yeast producing the capsid protein of red-spotted grouper nervous necrosis virus. Vet. Microbiol..

[334] Cho H.S., Seo J.Y., Park S.I., Kim T.G., Kim T.J. (2018). Oral immunization with recombinant protein antigen expressed in tobacco against fish nervous necrosis virus. J. Vet. Med. Sci..

[335] Gonzalez-Silvera D., Guardiola F.A., Espinosa C., Chaves-Pozo E., Esteban M.Á., Cuesta A. (2019). Recombinant nodavirus vaccine produced in bacteria and administered without purification elicits humoral immunity and protects European sea bass against infection. Fish Shellfish Immunol..

[336] Lin C.-F.F., Jiang H.-K.K., Chen N.-C.C., Wang T.-Y.Y., Chen T.-Y.Y. (2018). Novel subunit vaccine with linear array epitope protect giant grouper against nervous necrosis virus infection. Fish Shellfish Immunol..

[337] Luu V.-T., Moon H.Y., Hwang J.Y., Kang B.-K., Kang H.A. (2017). Development of recombinant *Yarrowia lipolytica* producing virus-like particles of a fish nervous necrosis virus. J. Microbiol..

[338] Yuasa K., Koesharyani I., Roza D., Mori K., Katata M., Nakai T. (2002). Immune response of humpback grouper, *Cromileptes altivelis* (Valenciennes) injected with the recombinant coat protein of betanodavirus. J. Fish Dis..

[339] Sommerset I., Skern R., Biering E., Bleie H., Fiksdal I.U., Grove S., Nerland A.H. (2005). Protection against Atlantic halibut nodavirus in turbot is induced by recombinant capsid protein vaccination but not following DNA vaccination. Fish Shellfish Immunol..

[340] Thiery R., Cozien J., Cabon J., Lamour F., Baud M., Schneemann A. (2006). Induction of a protective immune response against viral nervous necrosis in the European sea bass *Dicentrarchus labrax* by using betanodavirus virus-like particles. J. Virol..

[341] Wi G.R., Hwang J.Y., Kwon M.-G.G., Kim H.J.H.-J.J., Kang H.A., Kim H.J.H.-J.J. (2015). Protective immunity against nervous necrosis virus in convict grouper *Epinephelus septemfasciatus* following vaccination with virus-like particles produced in yeast *Saccharomyces cerevisiae*. Vet. Microbiol..

[342] Lin K., Zhu Z., Ge H., Zheng L., Huang Z., Wu S. (2016). Immunity to nervous necrosis virus infections of orange-spotted grouper (*Epinephelus coioides*) by vaccination with virus-like particles. Fish Shellfish Immunol..

[343] Chien M.-H.H., Wu S.-Y.Y., Lin C.-H.H. (2018). Oral immunization with cell-free self-assembly virus-like particles against orange-spotted grouper nervous necrosis virus in grouper larvae, *Epinephelus coioides*. Vet. Immunol. Immunopathol..

[344] Lai Y.-X.X., Jin B.-L.L., Xu Y., Huang L.J., Huang R.-Q.Q., Zhang Y., Kwang J., He J.-G.G., Xie J.-F.F. (2014). Immune responses of orange-spotted grouper, *Epinephelus coioides*, against virus-like particles of betanodavirus produced in Escherichia coli. Vet. Immunol. Immunopathol..

[345] Lan N.T., Kim H.J., Han H.-J., Lee D.-C., Kang B.K., Han S.Y., Moon H., Kim H.-J. (2018). Stability of virus-like particles of red-spotted grouper nervous necrosis virus in the aqueous state, and the vaccine potential of lyophilized particles. Biologicals.

[346] Sommerset I., Lorenzen E., Lorenzen N., Bleie H., Nerland A.H. (2003). A DNA vaccine directed against a rainbow trout rhabdovirus induces early protection against a nodavirus challenge in turbot. Vaccine.

[347] Vimal S., Abdul Majeed S., Nambi K.S.N., Madan N., Farook M.A., Venkatesan C., Taju G., Venu S., Subburaj R., Thirunavukkarasu A.R. (2014). Delivery of DNA vaccine using chitosan–tripolyphosphate (CS/TPP) nanoparticles in Asian sea bass, *Lates calcarifer* (Bloch, 1790) for protection against nodavirus infection. Aquaculture.

[348] Valero Y., Awad E., Buonocore F., Arizcun M., Esteban M.Á., Meseguer J., Chaves-Pozo E., Cuesta A. (2016). An oral chitosan DNA vaccine against nodavirus improves transcription of cell-mediated cytotoxicity and interferon genes in the European sea bass juveniles gut and survival upon infection. Dev. Comp. Immunol..

[349] Chen S.P., Peng R.H., Chiou P.P. (2015). Modulatory effect of CpG oligodeoxynucleotide on a DNA vaccine against nervous necrosis virus in orange-spotted grouper (*Epinephelus coioides*). Fish Shellfish Immunol..

[350] Vimal S., Farook M.A., Madan N., Abdul Majeed S., Nambi K.S.N., Taju G., Sundar raj N., Venu S., Subburaj R., Thirunavukkarasu A.R. (2016). Development, distribution and expression of a DNA vaccine against nodavirus in Asian Seabass, *Lates calcarifier* (Bloch, 1790). Aquac. Res..

[351] Huang J.N., Lin L., Weng S.P., He J.G. (2007). High expression of capsid protein of red-spotted grouper nervous necrosis virus in an avian cell line requires viral RNA2 non-coding regions. J. Fish Dis..

[352] Gaafar A.Y., Yamashita H., Istiqomah I., Kawato Y., Ninomiya K., Younes A.E., Nakai T. (2018). An oral vaccination method with the aid of Capsaicin against viral nervous necrosis (VNN). Fish Pathol..

[353] Lin C.-C., Lin J.H.-Y., Chen M.-S., Yang H.-L. (2007). An oral nervous necrosis virus vaccine that induces protective immunity in larvae of grouper (*Epinephelus coioides*). Aquaculture.

[354] Oh M.J., Gye H.J., Nishizawa T. (2013). Assessment of the sevenband grouper *Epinephelus septemfasciatus* with a live nervous necrosis virus (NNV) vaccine at natural seawater temperature. Vaccine.

[355] Boutier M., Ronsmans M., Ouyang P., Fournier G., Reschner A., Rakus K., Wilkie G.S., Farnir F., Bayrou C., Lieffrig F. (2015). Rational development of an attenuated recombinant Cyprinid herpesvirus 3 vaccine using prokaryotic mutagenesis and in vivo bioluminescent imaging. PLoS Pathog..

[356] Rouxel R.N., Tafalla C., Mérour E., Leal E., Biacchesi S., Brémont M. (2016). Attenuated infectious hematopoietic necrosis virus with rearranged gene order as potential vaccine. J. Virol..

